# Age‐friendly interventions in rural and remote areas: A scoping review

**DOI:** 10.1111/ajag.13101

**Published:** 2022-07-07

**Authors:** Jed Montayre, Jann Foster, Ivy Yan Zhao, Ariana Kong, Angela Y. M. Leung, Alex Molassiotis, Alana Officer, Christopher Mikton, Stephen Neville

**Affiliations:** ^1^ School of Nursing and Midwifery Western Sydney University Penrith New South Wales Australia; ^2^ World Health Organization Collaborating Centre for Community Health Services, School of Nursing The Hong Kong Polytechnic University Hong Kong SAR; ^3^ Department of Social Determinants of Health Division of Healthier Populations, World Health Organization Geneva Switzerland; ^4^ Nursing Department, School of Clinical Sciences Auckland University of Technology Auckland New Zealand

**Keywords:** ageing, healthy ageing, rural

## Abstract

**Objectives:**

In 2007, the World Health Organization published a guide on age‐friendly cities. However, little is known about interventions that have been implemented to promote age‐friendly communities in rural and remote areas. This paper presents the findings from a scoping review undertaken to locate available evidence of interventions, strategies, and programs that have been implemented in rural and remote areas to create age‐friendly communities.

**Methods:**

This scoping review used the Joanna Briggs Institute (JBI) methodology.

**Results:**

A total of 219 articles were included in this review. No intervention studies were referred to as ‘age‐friendly’. However, there were interventions (mostly healthcare‐related) that have been implemented in rural and remote areas with older people as participants. There were also non‐evaluated community programs that were published in the grey literature. This review identified the common health interventions in older people and the indirect relevance to the WHO age‐friendly framework domains in rural and remote contexts.

**Conclusions:**

The eight age‐friendly domains were not explicitly utilised as a guide in the development of interventions for older people in rural and remote settings. Implementation of age‐friendly interventions in rural and remote areas requires a multisectoral approach that is tailored to address the specific needs of individual communities. Age‐friendly interventions also need to consider socio‐ecological factors to adequately and holistically address community needs and ensure long‐term sustainability.

**Policy Impact**

The current scoping review identified the strengths and gaps in applying the WHO Age‐friendly Cities and Communities Framework to intervention studies conducted in rural and remote areas. To address the unique needs of older residents in these communities, policies should not only target a multisectoral team effort but also consider the drivers and motivation of individual communities in creating an age‐friendly environment.

**Practice Impact**

We have highlighted the importance of developing rural age‐friendly interventions tailored to and building on existing strengths and needs of specific communities. It is also necessary to formally evaluate existing practices of age‐friendly initiatives and future interventions at community level to ensure that the unique characteristics between and among rural communities are considered.

## 1 INTRODUCTION

The proportion of older people residing in rural and remote areas across the globe is expected to rise.^1^ In regions such as North America and Western Europe, the percentage of older people who live in rural areas ranges between 10% and 20%, while in some rural areas of Asia and sub‐Saharan Africa, this percentage could rise from 60% to over 80%.^2^ Factors that influence rural population ageing are complex and depend on a variety of social and economic conditions such as rates of urbanisation, fertility, life expectancy, age‐selective rural‐to‐urban migration of young individuals,^1^, ^3^ as well as inward migration (urban‐to‐rural) of older people for retirement. Although globalisation has resulted in some populations moving from rural regions to areas of faster economic growth,^3^ older people who continue to live rurally face complex challenges.

Rural and remote regions are characterised by smaller, dispersed populations at a distance from centres of services and amenities.^4^ The restricted economic scale in these areas has discouraged effective investment from governments in services and physical infrastructure such as public transportation.^3^ With relatively fewer economic opportunities, younger people in rural or remote areas tend to migrate to cities,^5^ increasing the proportion of older people in these areas. The outward migration of younger people can also reduce the accessibility and availability of support networks or services such as healthcare.^6^ These factors ultimately impact upon the well‐being of older people in rural areas compounded by poverty, social isolation, and declining health.^6^

There is also an increased focus on active ageing in the context of communities where older people live, since their experiences of ageing are dependent on the services and support structures that are available and accessible to them.^7^ In urban areas, the concept of age‐friendly cities was pioneered in consultation with the World Health Organization (WHO) and stakeholders from various countries.^8^ Age‐friendly cities are characterised by eight domains that promote active ageing: transportation; housing; social participation; respect and social inclusion; civic participation and employment; communication and information; community support and health services; and outdoor spaces and buildings.^8^ These eight domains may be identified as key areas that enable older people to live actively, securely, and in good health within the community.^8^ However, some communities where older people have spent most of their lives are becoming hostile and less supportive as places in which people can age in place.^9^ Therefore, the experience of ageing, by and large, is affected by the bi‐directional relationship between the ability to age in place and age‐friendly environments.

Since its inception, the WHO age‐friendly cities guide has been implemented flexibly by various cities, depending on local needs. Consequently, most published studies were located with an urban focus.^10^, ^11^ Little is known about the strategies and programs that have been implemented to promote age‐friendly communities in rural and remote areas. A scoping review was deemed an appropriate approach to locate and map the evidence relevant to this particular subject and to identify gaps. With this in mind, the aim of this scoping review was to map and summarise available evidence and key concepts, and to highlight interventions, strategies, and programs that have been implemented in rural and remote areas to promote age‐friendly communities.

## 2 METHODS

The review was conducted utilising the nine‐step process of the Joanna Briggs Institute (JBI) methodology for scoping reviews.^12^ The initial steps included protocol development, consultation with stakeholders to define terminologies and clarify overlapping concepts, and outlining the review aim and questions. The Preferred Reporting Items for Systematic reviews and Meta‐Analyses extension for Scoping Reviews (PRISMA‐ScR) was used to guide this review.^13^ As scoping reviews do not evaluate the quality of studies, both qualitative and quantitative studies, as well as a range of peer‐reviewed and non‐peer‐reviewed literature, were considered for inclusion. Scoping reviews also allow for a broad and an iterative process for developing the search strategy and screening based on the articles identified.^12^ A preliminary search of PROSPERO, MEDLINE, the Cochrane Database of Systematic Reviews, and JBI Evidence Synthesis was conducted and no systematic reviews or scoping reviews on the topic, either current or under way, were identified.

### 2.1 Eligibility criteria

Eligibility criteria were as follows:

- Published literature (academic and grey literature) that described an intervention including at least one domain outlined in the WHO age‐friendly cities guide^8^ in a rural or remote area; and
- Papers published in the last 10 years and in English, Chinese, French, and Spanish.

Studies published since 2010 were included, which coincided with the release of the WHO age‐friendly cities guide in 2007. In addition to the specific interventions, implementation barriers and facilitators of such interventions to provide future recommendations for the research in this area were considered. All literature that included older people (as defined by the study or paper) were included; this encompassed populations that were 50 years old and over to account for relative life expectancies across different countries and sub‐populations.^14^ Due to the complexities of defining ‘rural’ and ‘remote’ globally, this review took a broad approach and included any literature that was identified by the authors as ‘rural’ or ‘remote’. Literature that included older people living in urban or metropolitan areas but did not differentiate findings from rural areas were excluded.

### 2.2 Information sources

Literature from 2010 up to the 9 March 2021 were included for review. The databases and platforms searched included CINAHL, Scopus, ProQuest Central, PubMed, EBSCOHost, APA PsycInfo, Carin.info, and the European Network for Rural Development‐Rural Development Policy Projects database. Sources of unpublished studies/grey literature searched included EBSCOHost, ProQuest, government reports, websites, policy papers, and online newsletters. Additional records, particularly articles that were published by experts in age‐friendly research known to our network, were identified through a bibliographic hand search.

### 2.3 Search

A range of key terms, truncations, and the corresponding index terms (subject headings) related to the research aim were identified and used to develop a full search strategy. Variances in terminology and spelling across different countries were considered. These terms were translated from English to Spanish, Chinese, and French to avoid bias towards English‐speaking countries. This search strategy was adapted for each database or platform depending on the format of the search required. A research librarian was consulted to refine the search strategy. Keywords included age‐friendly, older people/persons, elder‐friendly, rural ageing, rural gerontology, ageing in place, and later life. Appendix S1 (Search Terms and Results) provides an example of a full electronic strategy for one database.

### 2.4 Selection of sources of evidence

**FIGURE 1:**
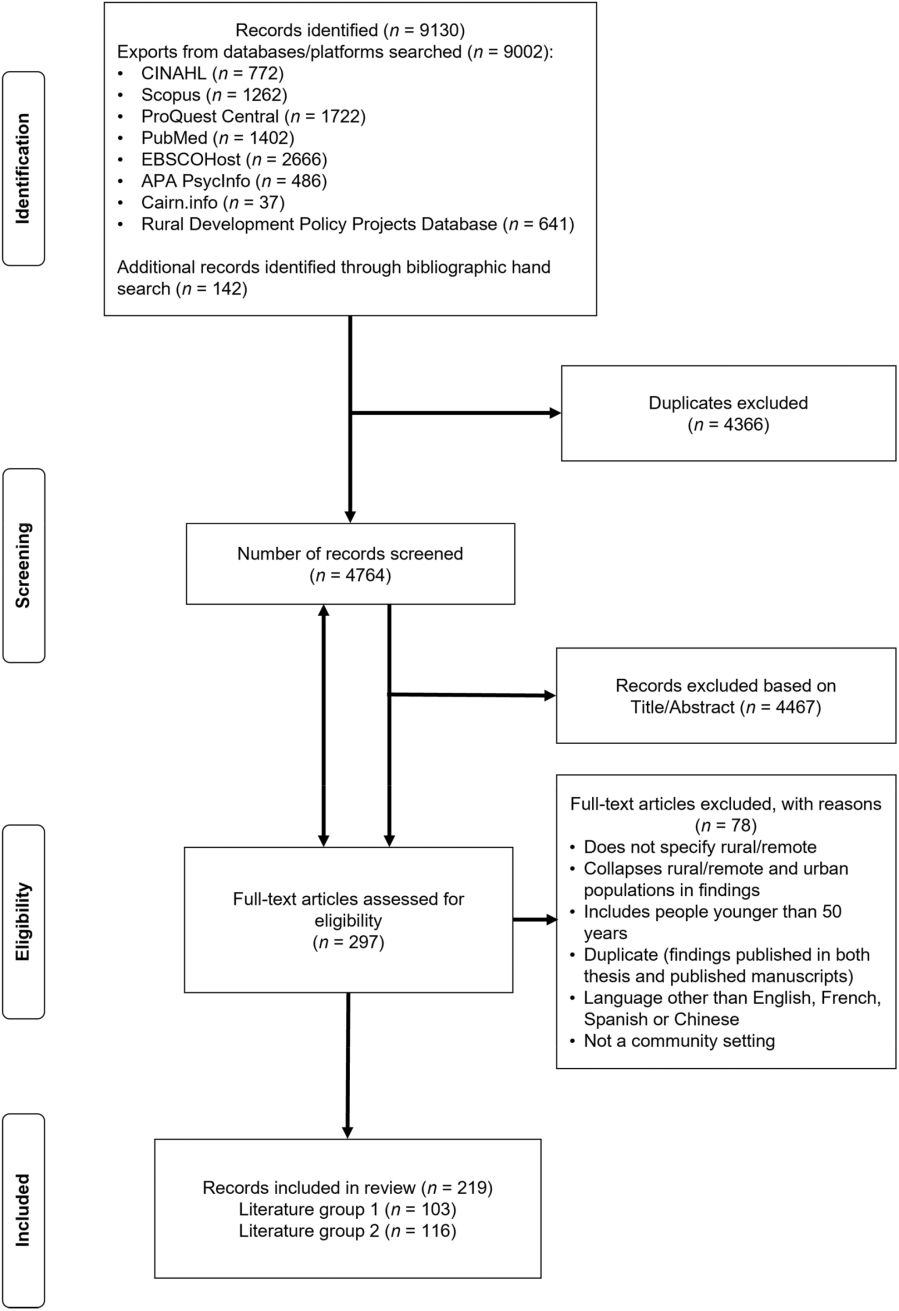
PRISMA diagram for scoping review

Following the search, all identified citations were exported and uploaded into Covidence Software for screening and review. Following the removal of all duplicates, the titles and abstracts were screened initially by at least two independent reviewers (the 1st, 3rd, and 4th authors) for assessment against the inclusion criteria. Where consensus was needed, other authors within the team who are experts in ageing research also reviewed the texts for inclusion. The full‐text article of each selected citation was assessed in detail against the inclusion criteria by at least two authors. Experts and topic consultants were consulted for verification and for additional information as part of the scoping review process. The results of the search and the study inclusion process are presented in PRISMA flow diagram (Figure 1).^13^

### 2.5 Data extraction

The data were extracted from the selected literature using the following headings: author/year; type of article; source; country; program/intervention; methodology; concept; context; justification; and outcome measures. Detailed summary tables and data characteristics are included in Appendix S1 Summary Table. After data extraction, emerging categories were identified (types of interventions; relevance to the WHO age‐friendly domains; intervention context; non‐evaluated programs and initiatives) and discussed with the team to create an a priori framework in which to group the studies. The literature was then coded based on the study's characteristics.

To ensure that a robust approach in categorising the interventions was undertaken, they were categorised according to their relevance to the eight age‐friendly domains. The team used an inter‐rater reliability exercise using case studies. This exercise involved the team members allocating the relevant age‐friendly domains to the study's intervention as per WHO's descriptions of the domains.^8^ Three case studies were selected. The review team extracted the age‐friendly domains from these three case studies independently. Then the overall agreement among the seven appraisers was calculated, and it was shown to be good with W = 0.69 (*p* < 0.05), confirming the inter‐rater reliability. The team was comprised of experts in ageing based in Australia, New Zealand, Hong Kong, and Europe. The team's expertise in ageing research included age‐friendly communities (the 1st, 2^nd^, and 9th authors), healthy ageing (the 3rd, 5^th^, and 6th authors), and aged care policies (all authors). Technical guidance was provided by two WHO Headquarters staff (the 7th and 8th authors). All team members had at least 7 years of experience undertaking research on ageing issues.

## 3 RESULTS

From the studies reviewed, no published age‐friendly intervention research undertaken in rural and remote areas was located. This was the main finding from this scoping review. However, we identified and reviewed evidence of mostly health‐related intervention studies on older people that were conducted in rural and remote settings linking to different domains of the WHO age‐friendly framework.^8^ The relevance of these interventions and corresponding outcomes to age‐friendly domains was examined and mapped as below.

### 3.1 Data characteristics

We identified two sets of literature. Literature group 1, a total of 103 studies, consisted of randomised controlled trials (*n* = 22), quasi‐experimental studies (*n* = 37), and a range of other quantitative, qualitative, and mixed‐methods study designs (*n* = 44). The summary tables for Literature group 1 (Appendix S1 Summary Table) have been separated into two tables; the first table (Literature group 1A) consists of studies that reported experimental and quasi‐experimental study designs while the second table (Literature group 1B) reports on studies with other methodologies. Studies that included both experimental and other methodologies have been repeated in both tables but with different information extracted.

Literature group 2 consisted of 116 non‐evaluated initiatives developed within local communities. These articles were published as grey literature on websites, in study protocols, and in community newsletters indexed in databases used for this scoping review. The term ‘interventions’ was used to describe the programs, strategies, and activities implemented for Literature group 1; it also was used to reflect the terminology used in many of the articles. As part of the evaluation of the included articles, they reported either a change in outcomes (outcome evaluation), described the implementation (process evaluation), or both. The term ‘initiatives’ was used to emphasise the non‐evaluated and non‐peer‐reviewed nature of Literature group 2.

The interventions in Literature group 1 were conducted across all six WHO Regions, with most coming from the region of the Americas (*n* = 54), the Western Pacific (*n* = 24), Europe (*n* = 12), and the South‐East Asia Region (*n* = 10). Only a few studies were from the African region (*n* = 2) or the Eastern Mediterranean (*n* = 1). The studies were carried out in 26 countries, with the majority being in Canada, Japan, South Korea, and the United States. Few intervention studies were conducted in middle‐ and low‐income countries.

### 3.2 Summary of themes

The results of this scoping review were mapped and categorised into four themes: (1) interventions with older people as participants, (2) the relevance of these interventions to the features and principles of the WHO Age‐friendly Cities and Communities framework, (3) the reasons for conducting interventions in rural and remote settings, and (4) non‐evaluated community initiatives. Table 1 provides a summarised context of the themes identified. The first three themes were identified from Literature group 1 and the fourth theme was derived from Literature group 2.

**TABLE 1:** Themes identified in this scoping review

Themes	Context
Interventions with older people as participants	Refers to interventions implemented in rural and remote areas that have been undertaken with older people as participants of the interventions. These were not identified as age‐friendly interventions.
Relevance to age‐friendly framework domains	Refers to the relevance of the interventions identified with the principles and features of the WHO Age‐friendly (AF) Cities framework.
Reasons for conducting interventions in rural and remote settings	Refers to the justification and rationale of researchers in implementing interventions in rural and remote settings.
Non‐evaluated programs and community initiatives	Refers to the community initiatives that were not evaluated at the municipal and local levels.

### 3.3 Theme 1: Interventions with older people as participants

In rural and remote settings, seven main types of interventions were identified, which recruited older people as participants. These types included Community Services (Pension); Community Services (Social); Community Services (Transportation); Education and Training; Exercise and Physical Activity; Health Promotion Programs, and Telehealth (Table 2).

**TABLE 2:** Literature group 1 by typology of interventions (*n* = 103)

Themes	Context
Interventions with older people as participants	Refers to interventions implemented in rural and remote areas that have been undertaken with older people as participants of the interventions. These were not identified as age‐friendly interventions.
Relevance to age‐friendly framework domains	Refers to the relevance of the interventions identified with the principles and features of the WHO Age‐friendly (AF) Cities framework.
Reasons for conducting interventions in rural and remote settings	Refers to the justification and rationale of researchers in implementing interventions in rural and remote settings.
Non‐evaluated programs and community initiatives	Refers to the community initiatives that were not evaluated at the municipal and local levels.

Health Promotion Programs (*n* = 44) were the most frequently reported interventions and were defined as interventions that fostered the abilities of older people to self‐manage their health and increase their knowledge about healthcare services in rural and remote areas. These programs were comprised of specific topics including health education and healthy practices. About two‐thirds (64%) of the studies that focussed on Health Promotion Programs utilised an experimental design.

Exercise and Physical Activity interventions (*n* = 23) measured specific health outcomes: for example in one Bangladeshi study, group exercises using pelvic floor and mobility techniques among older women led to improvements in continence.^14^ Others were measured against clinical outcomes related to physical function. A few Japanese studies (*n* = 3) that evaluated exercise programs measured objective clinical outcomes such as gait stability and motor function.^16^, ^17^, ^18^

Education and Training interventions (*n* = 15) were largely centred on managing health‐related needs by increasing literacy about specific diseases such as diabetes. The study by Ko et al.,^19^ for example, delivered an educational intervention about cataracts for older people and aimed to improve the knowledge, attitudes, and access of older people using eyecare services. Only one study provided training to improve the skills of older people to use the internet to access health information.^20^

Similar to Education and Training interventions, Telehealth interventions (*n* = 15) also tended to focus on specific diseases or conditions such as obesity, anxiety disorders, and diabetes.^21^, ^22^ Telehealth interventions were typically delivered via telephone, but in a few cases, video‐teleconferencing media was also used.

Only a few interventions related to Community Services (*n* = 6) that were neither healthcare nor health‐related were identified. Interventions that focussed on social groups established in the community and on transportation needs were limited. A shuttle bus service in rural areas in Canada and Ireland provided opportunities for older people to shop and participate in social activities in two studies.^23^, ^24^ Social clubs in the community generally focussed on process evaluations which tended to include interventions such as resident consultations and older people acting as volunteers.^25^, ^26^

### 3.4 Theme 2: Relevance to the WHO age‐friendly cities framework domains

The studies included in Literature group 1 were assessed according to their relevance to the eight domains of the age‐friendly cities framework (Table 3). None of the evaluated interventions included any which had been explicitly self‐identified as age‐friendly. The studies were classified against the descriptions and characteristics of the eight age‐friendly domains. This classification was guided by specific components found in the studies such as the aim, the nature of the intervention, and the justification for conducting the studies in rural and remote areas. Some interventions were relevant across multiple age‐friendly domains.

**TABLE 3:** Distribution of Literature group 1 studies by age‐friendly domains

WHO Age‐friendly Cities Domains Framework	No of Studies (*n* = 103)
AF: Transportation	4
AF: Housing	1
AF: Social participation	20
AF: Respect and social inclusion	9
AF: Civic participation and employment	6
AF: Communication and information	27
AF: Community support and health services	94
AF: Outdoor spaces and buildings	1

As most studies had a health‐related focus, most interventions were relevant to the community support and health services domain (*n* = 92). This was followed by communication and information (*n* = 27), and social participation (*n* = 20) (Table 3). Only two studies in Literature group 1 pertained to housing, outdoor spaces, and buildings in rural and remote contexts. Staniuliene and Januleviciene^27^ designed a domestic and social service within the homes of older people living in rural and remote areas; however, this intervention was aimed at improving health rather than housing. In another example, a case study by Zhenmian and Bixia^28^ described farming and agricultural economic opportunities for older people in rural Japan, which was relevant to civic participation and outdoor spaces and buildings.

Health promotion programs were classified as being relevant to seven out of the eight age‐friendly domains, with the exception being outdoor spaces and buildings. Some interventions were relevant to more than one age‐friendly domain. A health promotion program in Thailand, for example, involved volunteers visiting older people in their rural and remote homes with the aim of reducing depression and enhancing their quality of life.^29^ This Health Promotion Program intervention was relevant to Community Support and Health Services, Respect, and Inclusion, and encouraged Social Participation.

Exercise interventions were relevant to four of the eight age‐friendly domains. The majority of interventions relevant to the Social Participation domain were also categorised as Exercise and Physical Activity interventions. This categorisation is supported by individual studies in this review, where exercise interventions conducted in group classes provided opportunities to socialise and engage with others.^30^, ^31^

### 3.5 Theme 3: Rural and remote contexts

Most studies (*n* = 82) provided the contexts and justification for conducting these interventions in rural and remote areas. The significance of the interventions in rural and remote areas were highlighted in studies that had a focus on Health Promotion Programs, Education and Training, and Telehealth. The most common justifications for conducting the study in a rural or remote context were primarily due to limited access to health‐care services and due to an increasingly ageing population in rural communities.

Many studies recognised the limited access to healthcare services in rural and remote areas particularly for the treatment and management of common chronic conditions like diabetes.^32^, ^33^ There were studies that indicated the limited access to services such as palliative care, general health clinics, mental health, and specialty health services.^15^, ^34^, ^35^ Factors such as geographical isolation,^36^ financial constraints,^30^, ^37^ transportation issues,^18^, ^38^ and lack of rural health facilities and healthcare staff^39^, ^40^ were identified as complicating access to healthcare.

The population characteristics referred to the descriptions of residents in rural and remote areas. There were studies that described rural areas as having an increasing number of older residents.^41^, ^42^ Studies also identified older people in rural areas as having lower health literacy.^43^, ^44^ There was some evidence of low engagement with physical activity and health promotion programs,^45^, ^46^ and some studies reported that rural residents tended to be independent and did not recognise the need for help.^44^, ^47^ The studies included in this scoping review described how older rural residents commonly experience mental health issues such as depression^35^, ^48^ and chronic health conditions such as cataracts, diabetes, stroke,^19^, ^32^, ^33^, ^44^ and dementia.^49^ There was also evidence of increased hospitalisation and re‐hospitalisations.^50^ One study described the changing family and caregiving dynamics among families in rural communities, where adult children migrated to cities while older parents remained in rural areas.^51^

### 3.6 Theme 4: Non‐evaluated programs and community initiatives

The initiatives included in Literature group 2 (*n* = 16) were categorised based on the typology of the initiative (see Appendix S1 Summary Table). Some initiatives were categorised as having more than one typology. The majority of initiatives were identified under health and wellness (*n* = 47), followed by community projects (*n* = 19) and technology (*n* = 17). Some initiatives were centred around the built environment, specifically transport (*n* = 16), housing (*n* = 8) and infrastructure (*n* = 2). Only a small number were relevant to education and training (*n* = 4), provision of financial support (*n* = 4), volunteering (*n* = 3), or paid employment (*n* = 1). These programs and community initiatives were implemented at the municipal and/or local level in rural and remote settings as part of ongoing community development and service delivery activities. Due to the lack of evaluation of the outcomes, these were not able to be examined further in terms of their relevance to at least one domain outlined in the WHO age‐friendly cities guide.

## 4 DISCUSSION

The findings of this scoping review suggest that the Age‐friendly Cities and Communities framework and its eight domains were not utilised as a guide in the development of interventions for older people in rural and remote settings. The review found that interventions in rural and remote settings were primarily focussed on managing certain health conditions or facilitating health promotion activities that were deemed beneficial for the functional abilities of older people, such as exercise programs. These interventions did not explicitly aim to examine or explore the age‐friendliness of rural and remote communities.

The intervention studies reviewed only reported outcome measures related to the intervention and did not account for the impact of external factors such as the physical and social environment. For example, outcomes such as increased physical function can be influenced by multiple socio‐ecological factors. Exercise interventions that measured improvement of physical function in terms of gait, number of steps, and strength of extremities did not consider factors such as the ability of participants to interact socially while the interventions were implemented. In nearly all of the studies reviewed, the interventions did not consider the need of using the age‐friendly domains and principles of the Age‐friendly Cities and Communities framework in their design. Studies that reported on the built environment described these domains as either a barrier or facilitator rather than the focus of the intervention.

The implementation of the WHO Age‐friendly Cities and Communities framework in rural and remote settings poses several challenges, particularly when considering that such a framework was originally conceptualised using indicators that characterise metropolitan settings. The perceived ‘age‐friendliness’ of an environment is affected by inherent community features and characteristics such as degrees of rurality, topography and climate, size of the community, and its distance to an urban centre.^52^ Given that the framework was created based on the socio‐ecological characteristics of the urban environment, the diverse needs of different rural and remote communities may not be adequately addressed within the existing framework. One important issue for older people concerns transport. In an urban environment, for example, this might include walkability and the distance of transport stations or terminals to facilities or to their own residence.^53^ The inconvenient access to transportation in an urban environment is in stark contrast to the absence of public transportation services in rural and remote contexts.^54^ Older people in rural communities face changing and challenging environments, which require a whole‐of‐society approach to ensure that policies and environments address their needs.^7^

Despite the original intention of the Age‐friendly Cities framework, some characteristics and strategies that promote an age‐friendly city, such as stakeholder collaboration, government commitment, effective governance, and the involvement of older people in addressing social and physical environmental challenges, are also pertinent to rural and remote environments.^55^, ^56^ However, they may require additional considerations for rural and remote contexts. While the Age‐friendly Cities and Communities framework has been useful in advocating for best practice in promoting age‐friendly urban environments and has had a strong buy‐in from policy makers,^57^ our scoping review confirmed the gap and lack of evidence at a level of implementing interventions that are referred to as age‐friendly in rural and remote areas. A case study in rural Canada by McCrillis^58^ identified that a sense of community and connectedness was perceived as a strong factor in predicting the success and sustainability of an age‐friendly initiative. The study also found that communities that were fragmented, and had more diverse needs across the community, were less likely to be successful in implementing a long‐term sustainable intervention. Thus it is important that rural age‐friendly interventions are tailored to build on the existing strengths of a specific community where there is sufficient connectivity driven by community members rather than to utilise a macro‐level approach to implementation across multiple communities.

The Age‐friendly Cities and Communities framework was designed to guide practice and inform policies for interventions and initiatives that can be implemented at a community and societal level. However, evaluating interventions that address community‐ or societal‐level factors using experimental or quasi‐experimental designs presents challenges. Such challenges include finding sufficient units of analysis, assigning them randomly and finding appropriate control groups in non‐random studies.^59^, ^60^ Future research should focus on implementing interventions that consider the socio‐ecological factors that are relevant to older people within specific rural and remote communities. These factors should also include the age‐friendly framework domains related to the built environment, that is, outdoor spaces and buildings, transportation, and housing, given the paucity of evidence in this area. There is also a need to review evaluation practices of age‐friendly initiatives occurring at a community level to ensure that the unique characteristics of rural communities are considered.

This review has several strengths and limitations. The search was designed to be highly sensitive: multiple languages were considered for inclusion, databases for both peer‐reviewed and non‐peer‐reviewed literature were searched, and articles from key experts in age‐friendly approaches were also included. Furthermore, a comprehensive consultation process with global experts on ageing and age‐friendly cities was undertaken during the identification and screening of articles to define terms and concepts used in the review. The main unexpected limitation of the review was the paucity of literature that explicitly reported on using an age‐friendly framework to design an intervention. As a result, the categorisation of the literature based on age‐friendly characteristics was subjective, although the inter‐rater reliability exercise ensured that there was high agreement in the articles that were considered to have age‐friendly characteristics.

## 5 CONCLUSIONS

This scoping review identified a paucity of evidence that explicitly used the Age‐friendly Framework in rural and remote settings. The current landscape of interventions for older people in rural and remote settings focuses predominantly on health‐related, disease‐specific services and treatment. While interventions were mostly health‐focussed, there were no specific programs that tested or trialled interventions that are relevant to other age‐friendly domains such as the effects of transportation services, affordable housing schemes, and aspects related to the built environment and how that might impact upon health outcomes. Attention to socially‐focussed environmental factors within the Age‐friendly Framework is important when designing sustainable interventions in rural and remote settings. Moreover, there is the need to ensure that interventions within rural and remote settings are developed and implemented to address the needs of that community. Lastly, it is important to consider that communities also have assets and inherent capabilities that can be harnessed and optimised to create and ameliorate age‐friendly environments.

## CONFLICTS OF INTEREST

Dr Jed Montayre is an Associate Editor of the Australasian Journal on Ageing, and Professor Stephen Neville is a member of the Editorial Board of the same journal. Dr Ivy Yan Zhao, Professor Angela Y. M. Leung, and Professor Alex Molassiotis are employed by the World Health Organisation Collaborating Centre for Community Health Services in Hong Kong, SA. Alana Officer and Dr Christopher Mikton work for the World Health Organization in Geneva, Switzerland.

## Supporting information


Appendix S1

## Data Availability

The data that support the findings of this study are available in the supplementary file.
