# Supplementary material for: Age‐friendly interventions in rural and remote areas: A scoping review
Source: Australas J Ageing. 2022 Jul 7;41(4):490–500. doi: 10.1111/ajag.13101 (PMC10083949; doi:10.1111/ajag.13101)
Supplement: Supplementary file 1 — Appendix S1 [file AJAG-41-490-s001.docx]

**Supplementary Material**

***Supplementary File 1***

**Searching Terms and Results**

| **Database**  **Date** | **Search String** | **Results** |
| --- | --- | --- |
| CINAHL | **S1**  "age-friend*" OR "age-friendly program*" OR "age-friendly initiative*" OR "age-friendly environment*" OR "older people*" OR "older person*" OR "older adult*" OR "elder-friend*" OR "elder*" OR "50 years old and over" OR "60 years old and over" OR "rural ageing" OR "rural aging" OR "rural gerontology" OR "environmental gerontology" OR "liveable communit*" OR "soon to be old" OR "ageing in place" OR "later life" or “senior friendly” or “older population”  **S2**  (MM "Aging") OR (MM "Healthy Aging")  **S3**  (MM "Rural Population") OR (MM "Rural Health Services")  **S4**  rural or remote  **S5**  Program* or initiative or intervention or project or WHO domain or evaluat* OR checklist  **S6**  transport* OR access* OR infrastructure OR hous* OR home OR residence OR dwelling OR “social participation” OR “social involvement” OR “social engage*” OR “social active*” OR “social inclusion” OR respect OR “civic participation” OR “community participation” OR “community activ*” OR employ* OR job OR work OR communic* OR inform* OR “community support” OR “health service” OR “medical service” OR “outdoor space*” OR building OR recreation* OR “social determinants”  **S7** S1 OR S2  **S8** S3 OR S4  **S9** S5 AND S6  **S10** S7 AND S8 AND S9 | 772 |
| PubMed | (((Program*[Title/Abstract] OR initiative[Title/Abstract] OR intervention[Title/Abstract] OR project[Title/Abstract] OR WHO domain[Title/Abstract] OR evaluat*[Title/Abstract] OR checklist[Title/Abstract]) AND (transport* [Title/Abstract] OR access* [Title/Abstract] OR infrastructure [Title/Abstract] OR hous* [Title/Abstract] OR home [Title/Abstract] OR residence [Title/Abstract] OR dwelling [Title/Abstract] OR "social participation" [Title/Abstract] OR "social involvement" [Title/Abstract] OR "social engage*" [Title/Abstract] OR "social active*" [Title/Abstract] OR "social inclusion" [Title/Abstract] OR respect [Title/Abstract] OR "civic participation" [Title/Abstract] OR "community participation" [Title/Abstract] OR "community activ*" [Title/Abstract] OR employ* [Title/Abstract] OR job [Title/Abstract] OR work [Title/Abstract] OR communic* [Title/Abstract] OR inform* [Title/Abstract] OR "community support" [Title/Abstract] OR "health service" [Title/Abstract] OR "medical service" [Title/Abstract] OR "outdoor space*" [Title/Abstract] OR building [Title/Abstract] OR recreation* [Title/Abstract] OR "social determinants"[Title/Abstract])) AND (((((rural population[MeSH Terms]) OR (rural health services[MeSH Terms])) OR (rural health[MeSH Terms]))) OR (rural[Title/Abstract] OR remote[Title/Abstract]))) AND (((healthy aging[MeSH Terms]) OR (aging[MeSH Terms])) OR ("age-friend*"[Title/Abstract] OR "age-friendly program*"[Title/Abstract] OR "age-friendly initiative*"[Title/Abstract] OR "age-friendly environment*"[Title/Abstract] OR "older people*"[Title/Abstract] OR "older person*"[Title/Abstract] OR "older adult*"[Title/Abstract] OR "elder-friend*"[Title/Abstract] OR "elder*"[Title/Abstract] OR "50 years old[Title/Abstract] AND over"[Title/Abstract] OR "60 years old[Title/Abstract] AND over"[Title/Abstract] OR "rural ageing"[Title/Abstract] OR "rural aging"[Title/Abstract] OR "rural gerontology"[Title/Abstract] OR "environmental gerontology"[Title/Abstract] OR "liveable communit*"[Title/Abstract] OR "soon to be old"[Title/Abstract] OR "ageing in place"[Title/Abstract] OR "later life"[Title/Abstract] OR "senior friendly"[Title/Abstract] OR "older population"[Title/Abstract]))  Limits applied | 1402 |
| EBSCOHost  9/03/2021 | ("age-friend*" OR "age-friendly program*" OR "age-friendly initiative*" OR "age-friendly environment*" OR "older people*" OR "older person*" OR "older adult*" OR "elder-friend*" OR "elder*" OR "50 years old and over" OR "60 years old and over" OR "rural ageing" OR "rural aging" OR "rural gerontology" OR "environmental gerontology" OR "liveable communit*" OR "soon to be old" OR "ageing in place" OR "later life" OR "senior friendly" OR "older population") AND (rural OR remote) AND ((Program* OR initiative OR intervention OR project OR WHO domain OR evaluat* OR checklist)) AND ((transport* OR access* OR infrastructure OR hous* OR home OR residence OR dwelling OR "social participation" OR "social involvement" OR "social engage*" OR "social active*" OR "social inclusion" OR respect OR "civic participation" OR "community participation" OR "community activ*" OR employ* OR job OR work OR communic* OR inform* OR "community support" OR "health service" OR "medical service" OR "outdoor space*" OR building OR recreation* OR "social determinants"))  Search abstracts only  2010-present  Languages specified | 3478 (after limits applied)  Note that **2666** were exported since EBSCOHost automatically removes duplicates from exports |
| ProQuest | ab("age-friend*" OR "age-friendly program*" OR "age-friendly initiative*" OR "age-friendly environment*" OR "older people*" OR "older person*" OR "older adult*" OR "elder-friend*" OR "elder*" OR "50 years old and over" OR "60 years old and over" OR "rural ageing" OR "rural aging" OR "rural gerontology" OR "environmental gerontology" OR "liveable communit*" OR "soon to be old" OR "ageing in place" OR "later life" OR "senior friendly" OR "older population") AND ab(rural OR remote) AND ab(Program* OR initiative OR intervention OR project OR WHO domain OR evaluat* OR checklist) AND ab(transport* OR access* OR infrastructure OR hous* OR home OR residence OR dwelling OR "social participation" OR "social involvement" OR "social engage*" OR "social active*" OR "social inclusion" OR respect OR "civic participation" OR "community participation" OR "community activ*" OR employ* OR job OR work OR communic* OR inform* OR "community support" OR "health service" OR "medical service" OR "outdoor space*" OR building OR recreation* OR "social determinants") | 1722 |
| Scopus | ( TITLE-ABS-KEY ( "age-friend*" OR "age-friendly program*" OR "age-friendly initiative*" OR "age-friendly environment*" OR "older people*" OR "older person*" OR "older adult*" OR "elder-friend*" OR "elder*" OR "50 years old and over" OR "60 years old and over" OR "rural ageing" OR "rural aging" OR "rural gerontology" OR "environmental gerontology" OR "liveable communit*" OR "soon to be old" OR "ageing in place" OR "later life" OR "senior friendly" OR "older population" ) AND TITLE-ABS-KEY ( rural OR remote ) AND TITLE-ABS-KEY ( program* OR initiative OR intervention OR project OR who AND domain OR evaluat* OR checklist ) AND TITLE-ABS-KEY ( transport* OR access* OR infrastructure OR hous* OR home OR residence OR dwelling OR "social participation" OR "social involvement" OR "social engage*" OR "social active*" OR "social inclusion" OR respect OR "civic participation" OR "community participation" OR "community activ*" OR employ* OR job OR work OR communic* OR inform* OR "community support" OR "health service" OR "medical service" OR "outdoor space*" OR building OR recreation* OR "social determinants" ) ) AND PUBYEAR > 2009 AND ( LIMIT-TO ( LANGUAGE , "English" ) OR LIMIT-TO ( LANGUAGE , "Chinese" ) OR LIMIT-TO ( LANGUAGE , "Spanish" ) OR LIMIT-TO ( LANGUAGE , "Portuguese" ) OR LIMIT-TO ( LANGUAGE , "French" ) ) | 1262 |
| APA PsycInfo | ( "age-friend*" OR "age-friendly program*" OR "age-friendly initiative*" OR "age-friendly environment*" OR "older people*" OR "older person*" OR "older adult*" OR "elder-friend*" OR "elder*" OR "50 years old and over" OR "60 years old and over" OR "rural ageing" OR "rural aging" OR "rural gerontology" OR "environmental gerontology" OR "liveable communit*" OR "soon to be old" OR "ageing in place" OR "later life" or “senior friendly” or “older population” or (MM "Aging") OR (MM "Healthy Aging") ) AND AB ( (MM "Rural Population") OR (MM "Rural Health Services") or rural or remote ) AND AB ( Program* or initiative or intervention or project or WHO domain or evaluat* OR checklist ) AND AB ( transport* OR access* OR infrastructure OR hous* OR home OR residence OR dwelling OR “social participation” OR “social involvement” OR “social engage*” OR “social active*” OR “social inclusion” OR respect OR “civic participation” OR “community participation” OR “community activ*” OR employ* OR job OR work OR communic* OR inform* OR “community support” OR “health service” OR “medical service” OR “outdoor space*” OR building OR recreation* OR “social determinants” )  Limiters:  Abstracts only  2010-present  Languages specified: English, French, Chinese, Spanish | 486 |
| Cairn.info | («adapté à l’âge (des aînés)» OU «adaptés à l’âge (des aînés)» OU «qui réponde aux besoin des aînés» OU «qui répondent aux besoin des aînés» OU «favorable aux aînés» OU «favorables aux aînés» OU «adapté aux personnes âgées» OU «adaptés aux personnes âgées» OU «pour aînés» OU «programmes qui répondent aux besoin des aînés» OU « programmes favorables aux aînés» OU « programmes adaptés à l’âge (des aînés)» OU «initiatives qui répondent aux besoin des aînés» OU « initiatives favorables aux aînés» OU «initiatives adaptés à l’âge (des aînés)» OU «ville amie des aînés» OU «villes amies des aînés» OU «villes adaptées aux personnes âgées» OU «villes adaptées aux aînés» OU «aînés» OU «personnes âgées» OU «population vieillissante» OU «de 60 ans et plus» OU vieillissement dans les zones rurales» OU «vieillissement dans les régions rurales» OU «vieillissement dans les espaces ruraux» OU «vieillir en milieu rural» OU «gérontologie rurale» OU «vieillir sur place» OU «vieillir en place» OU «vieillissement chez soi» OU «vieillissement en place» OU «vieillissement sur place» OU «vieillir dans leurs logements»)  ET («zones rurales» OU «régions rurales» OU «espaces ruraux« OU «milieux ruraux» OU «régions éloignées» OU «régions reculées» OU «régions isolées» OU «zones éloignées» OU «zones reculées» OU «zones isolées») | 46 |
| Back search | Articles by the following authors were retrieved from PubMed (identified experts in age-friendly communities):   - Jeni Warburton - Rachel Winterton - Graham Rowles - Kieran Walsh - Mark Skinner - Vanessa Burholt - Thomas Scharf - Verena Menec | 142 |

***Supplementary File 2***

**Literature 1A Summary Table**

|  | **Authors & Year** | **Type** | **Country** | **Study Aims** | **Study Design** | **Justification for setting** | **Intervention** | **Typology of Intervention** | **Outcomes** | **Type of Evaluation** |
| --- | --- | --- | --- | --- | --- | --- | --- | --- | --- | --- |
| 1 | Arnold et al. (2016) | Journal Article | United States | Evaluate if Fecal Occult Blood Test rates could be sustained over 3 years in rural US | Randomized controlled trial | In previous screening programs, fecal occult blood test screening is low in individuals from rural areas | Enhanced care; educational sessions; nurse support (combination) | Education and Training | FOBT screening rates were not sustained with any of the three interventions | Outcome |
| 2 | Batsis et al. (2021a) | Journal Article | United States | Evaluate the feasibility, acceptability, and effectiveness of integrating a wearable Fitbit device into a high-touch, multicomponent weight loss intervention at a local community aging center | Quasi-experimental (Pilot feasibility) | The program serves a predominantly rural catchment area where 14.5% of population were >65 years (compared to 11% in national average) | Individualized physical and nutrition program with use of Fitbit | Exercise and Physical Activity | Multicomponent obesity intervention (using a wearable device) was feasible and acceptable to older adults with obesity; significant improvement observed across physical function, late life function and subjective physical and mental scores | Outcomes & process |
| 3 | Batsis et al. (2021b) | Journal Article | United States | Determine the feasibility, acceptability and preliminary outcomes of an integrated technology-based health promotion intervention in rural-living, older adults using remote monitoring and synchronous video-based technology | Quasi-experimental | 78% of veterans in Maine lived in rural areas, and 2.1% in highly rural areas | An integrated technology-based health promotion intervention | Telehealth | Feasible and acceptable to older adults with obesity; significant improvements in weight loss and physical function | Outcomes & process |
| 4 | Blocker (2019)* | Dissertation/  Thesis | United States | Increase physical activity among middle-aged and older adults living in rural Kansas, increase Alzheimer's disease knowledge through risk reduction education, and determine the unique barriers to physical activity and exercise in this rural Kansas cohort | Quasi-experimental | Insufficient accessibility of healthcare resources for older adults who live in rural areas | 10-week community-based education and exercise intervention program | Exercise and Physical Activity | Healthy lifestyle outcomes significantly improved for education & exercise group; other outcomes were not significantly improved for the education & exercise group compared to control or education-only group | Outcomes |
| 5 | Brenes et al. (2015) | Journal Article | United States | Examine the effects of telephone-delivered cognitive behavioral therapy (CBT) compared with telephone-delivered nondirective supportive therapy (NST) in rural older adults with generalized anxiety disorder | Randomized clinical trial | Older adults in rural areas are likely to have lower health literacy, which can impact on health decisions and overall health | Telephone-delivered cognitive behavioral therapy consisted of as many as 11 sessions (9 were required) | Telehealth | Significantly greater decline in worry severity, generalized anxiety disorder symptoms and depressive symptoms among participants in the telephone-delivered cognitive behavioral therapy group | Outcome |
| 6 | Chang et al. (2020) | Journal Article | Taiwan | Investigate the influence of fitness trackers with different goal setting strategies for older adults on physical activity | Randomized controlled trial | No justification for rural setting | Individualized goal setting based on their individual physical activity level | Exercise and Physical Activity | Compared to the universal group, the individual group experienced immediate effects within a short period of two weeks. However, there was no significant difference between the two groups | Outcome |
| 7 | Chueh et al. (2012) | Conference abstract/  proceeding | Taiwan | Evaluate the effects of auricular acupressure (AA) on sleep disturbance on elderly aboriginal women | Quasi-experimental | Older adults are physically inactive, and in rural areas; there is limited availability of physical activity programs | Auricular acupressure | Health Promotion Programs | Improved sleep disturbance | Outcome |
| 8 | Crandall et al. (2019) | Journal Article | United States | Evaluate the impact of a game-centered mobile app (Bingocize®) on older adults' knowledge, skill, and confidence for managing aspects of their healthcare | Randomized controlled trial | No justification for rural setting | Health education and an exercise component in a group setting with use of app | Education and Training | Increased skills, confidence and knowledge of health | Outcome |
| 9 | de Batlle et al. (2020) | Journal Article | Spain | Assess the acceptability, usability, and satisfaction of an mHealth-enabled integrated care model for complex chronic patients in both patients and health professionals | Quasi-experimental (Feasibility) | Many rural adults with diabetes have limited access to diabetes educators; telemedicine is a feasible and acceptable approach to providing services. | Wearable device and integrated care model | Health Promotion Programs | Patient acceptability and usability were high but staff acceptability and usability were low and average respectively, although actual use of technology was high | Outcomes & process |
| 10 | DiNapoli et al. (2017) | Journal Article | United States | Examine the effects of home-delivered cognitive-behavioral therapy (CBT) for depression on anxiety symptoms in an ethnically diverse, low resource, and medically frail sample of rural, older adults | Randomized controlled trial | Unmet treatment needs among rural adults | Cognitive behavioral therapy | Health Promotion Programs | Improved quality of life and reduction in psychological symptoms | Outcome |
| 11 | Dongre et al. (2012) | Journal Article | India | Evaluate the effect of a community-managed palliative care program on perceived quality of life in the elderly in the project villages in rural Tamil Nadu. | Randomized controlled trial | No justification for rural setting | Community-Managed Palliative Care Program | Health Promotion Programs | The model of “community-managed” palliative care program improved perceived physical quality of life and psychological support among the elderly; no impact on social relationship and environment domains | Outcome |
| 12 | Dumitrache et al. (2017) | Journal Article | Spain | Explore the effectiveness of an intervention program aiming at improving quality of life in a group of community-dwelling older adults living in a depopulated rural area in Orense, Galicia, Spain | Quasi-experimental (Pre-post) | Ageing has not been studied extensively, unbalanced distribution of resources among rural older adults compared to urban adults; Spanish ageing policies do not give specific recommendations about older people | Workshops aimed at improving cognitive and physical function | Health Promotion Programs | Intervention reduced risk of cognitive impairment; participants believed that opportunity for leisure activities increased which also corresponded with increased in perception of mental health | Outcome |
| 13 | Dye et al. (2018) | Journal Article | United States | Pilot test a model to reduce hospital readmissions and emergency department use of rural, older adults with chronic diseases discharged from home health services (HHS) through the use of volunteers | Quasi-experimental (Pilot feasibility) | Rural populations experience poorer health outcomes compared to urban counterparts due to lower socioeconomic status, reduced access to health services, and incidence of chronic disease; rural adults are frequently readmitted for the same health conditions | Use of volunteer community members who were trained as health coaches | Health Promotion Programs | Program participants were able to monitor and track their chronic health conditions, make positive lifestyle changes, reduce incidence of falls, pneumonia and flu; No significant differences in ED/hospital admission rates | Outcome |
| 14 | Elder et al. (2016)* | Journal Article | United States | Examine satisfaction with and outcomes of a real-time Internet-based group exercise program for older adults | Quasi-experimental | Limited access to health care services in rural areas; physical activity can be partially dependent on access to safe, low-cost, inviting environments; limited opportunities for indoor physical activity | 10-week distance group exercise program | Exercise and Physical Activity | Intervention found to be valuable by participants; significant improvement in physical function | Outcomes & process |
| 15 | Ford et al. (2017) | Journal Article | United States | To evaluate the effectiveness of a quality improvement intervention to increase delivery of 2 evidence-based health promotion workshops, Stepping On and Chronic Disease Self-Management Program (CDSMP), in rural communities. | Cluster-randomized wait-list control group design | Higher proportion of older people in rural setting; difficult to engage older people in rural areas with health-promotion programs; inadequate financial or staff resources to implement these programs | Training and coaching county aging unit staff to provide workshops | Health Promotion Programs | Significant reduction in falls, decline in emergency department visits | Outcome |
| 16 | Fouladbakhsh et al. (2011) | Journal Article | United States | Evaluate the effects of an intervention to teach older adults in a rural community about the use of nondrug therapies for self-treatment of pain | Quasi-experimental | Limited access to medical care and financial resources mean that rural residents may prefer to opt for self-treatment in health care; higher prevalence of pain and non-drug therapies for management with less information/access about these therapies | Education | Education and Training | Significant increase in the use of non-drug treatments and a decrease in pain-related distress and pain scores | Outcome |
| 17 | Giesbrecht et al. (2015) | Journal Article | Canada | Determine the acceptability and feasibility of administering an mHealth wheelchair skills training program safely and effectively with two participants of different skill levels | Quasi-experimental (Pre-post feasibility) | Need for alternative and innovative electronic and mobile technology strategies to deliver health-related services in rural settings | Training and home education program | Education and Training | Program was acceptable and feasible, some initial issues with configuration of program | Outcomes & process |
| 18 | Haque et al. (2020) | Journal Article | Bangladesh | Pelvic floor and mobility exercises were shown to be effective in managing incontinence in a cluster‐randomized trial (CRT) of village women aged 60 to 75 years in Bangladesh. The present analysis examines continence 12 months after the CRT and exercise program implementation with village paramedics as preceptors. | Quasi-experimental (Pre-post) | Few resources at community clinics in rural area to meet needs | Group exercise program | Exercise and Physical Activity | Improvement in continence | Outcome |
| 19 | Hockin (2013) | Dissertation/Thesis | United States | Evaluate the relationship between cognitive training (CT) and problem solving in older adults 12 to 24 months post CT intervention | Quasi-experimental (Hybrid, ex post facto) | No justification for rural setting | Cognitive training | Health Promotion Programs | No significant relationships between cognitive training or demographic variables and problem solving were found | Outcome |
| 20 | Hosseini et al. (2013) | Journal Article | Iran | Define the effect of nurse home visits on self-care self-efficacy of the elderly in rural areas | Quasi-experimental (randomized, pre-post) | Proportionally, there are more older adults living in rural areas compared to urban areas; importance of health-promoting self-care behaviors in increasing quality of life in the elderly | Five home visit programs and one group session by a nurse during 6 weeks, and included two different sections of education and nursing interventions administered based on needs assessment and determination of the tasks for the clients and their families. | Health Promotion Programs | A significant difference was seen in the mean total scores of self-care self-efficacy and its subscales | Outcome |
| 21 | Hsu et al. (2018) | Journal Article | Taiwan | Implement and evaluate a cross-disciplinary intervention program using two approaches for community-based older adults in Taichung, Taiwan | Quasi-experimental | No justification for rural setting | Cross-disciplinary intervention program using personal-and-digital approaches aimed at promoting healthy ageing | Telehealth | Among rural population, the ability to search for health information improved, as did self-rated health. | Outcome |
| 22 | Hu et al. (2017) | Journal Article | China | Evaluate the effectiveness of a synthetic intervention model aimed at preventing type 2 diabetes and controlling plasma glucose, body weight and waist circumference in elderly individuals with prediabetes in rural China | Randomized controlled trial | Previous interventions for diabetes have been conducted in resource-intensive areas; however, these may not work in rural areas with social, economic and cultural differences | Lifestyle health promotion intervention for diabetes (synthetic intervention model consisted of lifestyle education, counselling, self-monitoring of blood glucose, and setting up a Health Each Other Group) | Health Promotion Programs | Intervention contributed to weight loss and decrease in fasting glucose | Outcome |
| 23 | Izquierdo et al. (2010) | Journal Article | United States | Examine the changes in waist circumference (WC) and body mass index (BMI) in older adults enrolled in a diabetes telemedicine program | Randomized controlled trial | Potential for telemedicine to deliver care to individuals in rural areas with diabetes who have poor access to health care | Diabetes telemedicine program | Telehealth | The program improved self-reported diet/exercise related knowledge and practices; changes were associated with improved physical measure (waist circumference and BMI) | Outcome |
| 24 | Jang et al. (2018a) | Journal Article | South Korea | Evaluate the effectiveness of a 6-month multicomponent intervention on physical function in socioeconomically vulnerable older adults in rural communities. The study also evaluated the effectiveness of the intervention on frailty and other geriatric syndromes, sustained benefit at 12 months, and baseline characteristics associated with poor response. | Quasi-experimental (Designed-delay pre-post) | Prevalence frailty especially high among older adults who live in rural areas, and since there are fewer facilities/resources, they are at higher risk of functional decline; effectiveness/feasibility of interventions addressing this have not been explored that extensively | Multicomponent program for physical and mental wellbeing | Health Promotion Programs | Improved physical function, frailty, sarcopenia, depressive symptoms and nutritional status. | Outcome |
| 25 | Jang et al. (2018b) | Journal Article | South Korea | We aimed to evaluate whether a wearable device and mobile-based intermittent coaching or self-management could increase physical activity and health outcomes of small groups of older adults in rural areas | Quasi-experimental (Feasibility) | Older adults living in rural areas have limited availability for health care services and tend to have lower socioeconomic status, educational level, higher prevalence of living alone, multi-morbidity, frailty and disability | Wearable device and mobile-based coaching and self-management | Health Promotion Programs | The Smart Walk program improved physical fitness, anthropometric measurements and geriatric assessment | Outcome |
| 26 | Jeon et al. (2014) | Journal Article | South Korea | Devise a recurrent fall prevention program for elderly women in rural areas | Randomized controlled trial | Older people who live in rural communities have less access to treatment and education for their recovery of physical and psychological impairments caused by falling compared to urban adults | Physical activity and education - fall prevention program | Exercise and Physical Activity | Improves muscle strength, endurance, balance and psychological outcomes | Outcome |
| 27 | Jindo et al. (2016) | Journal Article | Japan | Compare the effect on LEPF (lower extremity physical function) between an exercise intervention with and without the use of a pedometer | Quasi-experimental | No justification for rural setting | Fall-prevention exercise | Exercise and Physical Activity | Some improvement in physical function, findings suggest that the use of a pedometer is useful to improve LEPF | Outcome |
| 28 | Jindo et al. (2017) | Journal Article | Japan | Investigate how daily life physical activity modulates the effects of an exercise program on LEPF (lower extremity physical function) | Quasi-experimental | No justification for rural setting | Fall-prevention program using exercise | Exercise and Physical Activity | Square-stepping exercise can improve lower extremity physical function | Outcome |
| 29 | Jo et al. (2016) | Journal Article | South Korea | Apply the “Happy Together” integrated cognitive behavior program for elderly people with dementia who are living at home | Quasi-experimental | Elderly people with dementia tend to be cared for at home in rural areas, with family typically being the main caregiver | Integrated cognitive behavioral training | Health Promotion Programs | Significant change with activities of daily life and burden for caregivers | Outcome |
| 30 | Kawamoto et al. (2015) | Journal Article | Japan | Determine whether baseline arterial stiffness is independently associated with improvement in functional abilities after exercise training | Quasi-experimental (Pre-post) | No justification for rural setting | 12-week Nordic walking program | Exercise and Physical Activity | Baseline brachial ankle stiffness may be a predictor of functional ability after exercise | Outcome |
| 31 | Kim et al. (2017) | Journal Article | South Korea | Evaluated the effectiveness of telemedicine in relation to cognitive changes in patients with dementia | Quasi-experimental (non-randomized controlled trial) | No justification for rural setting | Telemedicine services | Telehealth | Cognitive decline was significantly lower in the telemedicine group for the less severe initial cognitive performance subgroup than more severe cognitive performance subgroup | Outcome |
| 32 | Ko et al. (2021) | Journal Article | Myanmar | Measure the effect of door-to-door eye health education in improving knowledge and attitude toward cataracts and the use of eyecare services among the elderly residing in a rural area of Naypyitaw Union Territory, Myanmar | Quasi-experimental | High prevalence of eye diseases in identified rural town, with no recorded cataract intervention | Cataract education delivered at home | Education and Training | Significantly increased knowledge and attitude toward cataracts and the use of eyecare services among the elderly with cataracts. | Outcome |
| 33 | Köhler et al. (2014) | Journal Article | Germany | Evaluate an already existing regional dementia network | Randomized controlled trial | Rapidly aging population with limited local medical care | Regional dementia network | Health Promotion Programs | Interdisciplinary regional network provides significant advantages with getting medication and access to appropriate specialists; No significant difference in quality of life nor overall effects or treatment by time effects | Outcome |
| 34 | Krajnik (2018) | Dissertation/  Thesis | United States | Investigate the feasibility of providing theory-based computer and Internet skills training to rural-dwelling older adults and explore the effects on their ability to locate and evaluate online health information | Quasi-experimental (Pre-post feasibility) | The computer and Internet health information concerns of the rural older adults have not been explored, even though they have limited access to healthcare providers and tend to experience poorer health | Educational program around computer and Internet health training | Education and Training | Found to be feasible and improved knowledge and self-perceptions of electronic health literacy | Outcomes & process |
| 35 | Kume et al. (2019) | Journal Article | Japan | Examine whether the multicomponent exercise program based on an independent home-training can become effective strategy for Japanese older people in a depopulated rural region | Quasi-experimental | Very high >65 population in rural city (44% in 2017), and the need for exercise among older adults especially due to limited transport access | Multicomponent exercise program | Exercise and Physical Activity | Exercise program based on home-training can be effective for enhancements of the gait ability and the executive function for older individuals in a depopulated rural region | Outcome |
| 36 | Lembeck et al. (2019) | Journal Article | Denmark | Determine whether discharge planning including a single follow-up home visit reduces readmission rate | Randomized controlled trial | Hospitalizations due to ambulatory care sensitive conditions are greater among people with certain sociodemographic characteristics (suggestive that rural may be a characteristic of interest) | Follow up home visit following hospital discharge (hospital at home intervention) | Health Promotion Programs | No effect on readmission rates nor on any secondary outcomes | Outcome |
| 37 | Lin et al. (2015) | Journal Article | Taiwan | Tests the effectiveness of a diet and exercise program on the MetS biomarkers in older community residents with metabolic syndrome | Quasi-experimental | Few studies have studied the effect of a combined diet and exercise program on managing metabolic syndrome (MetS) in individuals 65 years and older and living in Taiwan’s rural areas | Combined diet and exercise program | Health Promotion Programs | Intervention group had significantly improved anthropometric measurements and biomarkers | Outcome |
| 38 | MacIntyre et al. (2019) | Journal Article | Canada | Improve osteoporosis care and decrease bone fracture risk in a population of patients 65 years of age and older | Quasi-experimental | No justification for rural setting | Invitation to self-refer for osteoporosis and nurse-led intervention | Health Promotion Programs | Self-referral program resulted in an increase in the number of people who tested for bone mineral density; proportion of low-risk participants recently tested higher than those previously tested for bone mineral density | Outcome |
| 39 | Mangalvedhe et al. (2017) | Journal Article | India | The present study was conducted to know the impact of Otago Exercise Intervention on risk of falls in the community in a village in Mysore district | Quasi-experimental (Pre-post) | No justification for rural setting | Otago exercise intervention | Exercise and Physical Activity | Exercise intervention effective in reducing risk of falls, improvement in physical function | Outcome |
| 40 | Matsubayashi et al. (2016) | Journal Article | Japan | This study examined whether low-frequency group exercise improved the motor functions of community-dwelling elderly people in a rural area when combined with home exercise with self-monitoring | Quasi-experimental | Reduced human resources in rural areas; issues with good transportation network in these areas so an intervention with few should be explored | Group exercise and home exercise | Exercise and Physical Activity | Intervention improved physical function | Outcome |
| 41 | McMahon et al. (2016) | Journal Article | United States | To assess the feasibility of a new intervention, Ready~Steady, in terms of demand, acceptability, implementation, and limited efficacy. | Randomized controlled trial | No justification for rural setting | Program providing social support and falls-prevention, inclusive of an app | Exercise and Physical Activity | Acceptable to population and good implementation fidelity. Improved physical function | Outcomes & process |
| 42 | Naik et al. (2012)* | Journal Article | United States | Test the acceptability, feasibility and preliminary outcomes of a telephone-delivered coaching intervention for rural-dwelling older adults with uncontrolled diabetes and comorbid, clinically significant depressive symptoms. | Quasi-experimental | Treatment options for diabetes and depression are rarely available for patients in rural settings | Telehealth & diabetes (disease-specific) | Telehealth | Participants responded positively to the collaborative goal setting and action-planning; clinically significant improvements observed to outcomes relating to diabetes and depression | Outcomes & process |
| 43 | Nott et al. (2019) | Journal Article | Australia | This pilot trial examined the feasibility and effectiveness of “Ageing Well,” a community‐based program for improving cognitive skills and mobility of rural older people. | Quasi-experimental (Non‐randomized, wait‐list–controlled pilot trial) | Geographic isolation; dispersed population and vulnerability to economic restructuring means that interventions for healthy aging need to take a multidimensional approach; lack of studies including older people living in regional/rural communities | Dual‐tasking activities to improve motor and cognitive abilities | Health Promotion Programs | Ageing Well program is feasible, safe and acceptable; data supports some effectiveness | Outcomes & process |
| 44 | Oh et al. (2020a) | Journal Article | South Korea | Examine the feasibility and effectiveness of a village-based intervention for depression targeting older adults living in rural areas | Randomized controlled trial | Limited access to treatment for late-life depression among older people living in rural areas | Case management and group-based activities for depression | Health Promotion Programs | Intervention did not reduce depressive symptoms in the older population overall, which was probably due to the inclusion of large numbers of healthy elderly individuals; intervention lowered the risk of progression to severe depression among at-risk older adults, and increased the social network more than five-fold | Outcome |
| 45 | Oh et al. (2020b) | Journal Article | South Korea | This study investigated the effectiveness of an integrated intervention program combining self-directed home-based resistance training with health education for older adults with knee osteoarthritis living in a rural area | Randomized controlled trial | Impact of integrated intervention programs among people with osteoarthritis in rural areas not known | Health education and home-based resistance training | Education and Training | Improvement in mobility/physical function | Outcome |
| 46 | Ohta et al. (2021)* | Journal Article | Japan | Study hypothesized that social cognitive theory (SCT)-based educational interventions for healthcare participation can improve the self-efficacy of older rural citizens in participating in their health management without any difficulties | Quasi-experimental | People in rural areas tend to have lower health literacy skills, social norms mean that people consider living without help virtue (stronger among older people) | Social cognitive theory (SCT)-based educational intervention | Education and Training | Social cognitive theory-based educational interventions can positively impact self-efficacy in healthcare participation | Outcome |
| 47 | Olakehinde et al. (2019) | Journal Article | Nigeria | Investigate the feasibility and clinical impact of a psychosocial intervention, Cognitive Stimulation Therapy (CST), to help manage dementia in a rural setting in Nigeria | Quasi-experimental (Pre-post feasibility) | No justification for a rural setting | Cognitive Stimulation Therapy (CST) | Health Promotion Programs | Cognitive Stimulation Therapy is feasible in this setting; Significant improvements in cognitive function, quality of life (physical, psychosocial and environmental domains), physical function, neuro-psychiatric symptoms and carer burden | Outcomes & process |
| 48 | Paschoa & Ashton (2016)* | Journal Article | United States | Evaluate a group exercise program for older adults living in a rural community | Quasi-experimental | Physical inactivity is higher among rural populations, this may be attributed to: 1) natural social networks of older adults may not reinforce an active lifestyle; 2) older adults fear exercising outside due to unsafe neighbourhoods or natural environments; 3) lack of knowledge of the importance of exercising to improve quality of life, health status, daily functioning, health care costs and knowledge on how to exercise safely | Exercise programs focused on reducing sedentary behavior; improved physical function and participants were satisfied with the program | Exercise and Physical Activity | Improved knowledge and physical function; participants were satisfied with the program | Outcome & process |
| 49 | Pesut et al. (2017)* | Journal Article | Canada | Pilot a nurse-led navigation service to provide early palliative support for rural older adults and their families living at home with advancing chronic illness | Quasi-experimental (pilot) | Few available services to support rural older people receiving palliative care, even though they require more transition compared to urban residents; rural communities also have the capacity to provide high quality care as there are both personal and professional relationships co-existing | Palliative care support delivered at home | Health Promotion Programs | Intervention is a promising intervention to meet the needs of population | Outcome & process |
| 50 | Rachasrimuang et al. (2018) | Journal Article | Thailand | Evaluate the effectiveness of home visits programme by a youth volunteer on the health-related quality of life and depression among elderly persons living in a rural community | Cluster randomized controlled trial | No justification for rural setting | Trained youth (Grades 6-9) volunteers to visit older people in their homes | Health Promotion Programs | Improved health and depression score | Outcome |
| 51 | Rana et al. (2010) | Journal Article | Bangladesh | To examine changes in self-reported arthritis-related illness and self-rated health as a result of a health education intervention, and the association between self-reported arthritis-related illness and self-rated health | Quasi-experimental | No justification for rural setting | Home-based physical activities, health advice | Exercise and Physical Activity | Those who were compliant to health advice were more likely to report improvement in arthritic-related illnesses; those who were literate and non-poor were more likely to report positive health | Outcome |
| 52 | Rizkalla (2015) | Dissertation/  Thesis | Canada | 1. Develop a novel collaborative approach to improving access to cognitive training in rural settings; 2. Evaluate the efficacy of a multicomponent cognitive training program for improving cognition in normal elderly persons | Randomized controlled trial | Lack of access to specialty services for rural older adults in Canada | Three training modules to improve cognitive function | Education and Training | Improved executive function and memory but not psychosocial ability | Outcome |
| 53 | Sarkar et al. (2017) | Journal Article | India | Assess the impact of attendance at a community-based daycare center in rural Puducherry, India, on depression, cognitive impairment (CI) and quality of life of the elderly | Quasi-experimental (Pre-post) | Majority of elderly population live in rural areas; rural elderly were worst affected by changing family structure in Indian villages as well as migration of younger people to urban areas which makes them more vulnerable to access social services and health facilities. | Problem-solving therapy (psychological therapy) over 4-5 sessions | Health Promotion Programs | Attendance at daycare center reduced risk of depression by half; improved social domain of quality of life score | Outcome |
| 54 | Schweickert et al. (2011) | Journal Article | United States | Evaluate the effectiveness of delivering stroke education to elderly individuals through telehealth versus in person stroke prevention education methods | Quasi-experimental (Pre-post) | Stroke is prevalence among elderly peoples; however, education is difficult to deliver in rural/remote regions | Mixed telehealth and in-person education | Telehealth | Telehealth stroke education is feasible; there were no between-group differences in changes in knowledge or likelihood in making behavioral changes but changes were observed pre-post intervention for intervention group | Outcomes & process |
| 55 | Scogin et al. (2014) | Journal Article | United States | Examine the effects of home-delivered cognitive-behavior therapy (CBT) on depressive symptoms among rural, diverse, and vulnerable older adults | Randomized controlled trial | High number of older adults living in rural areas and risk of mental health in this group; paucity of research of cognitive behavioral therapy | Cognitive behavior therapy | Health Promotion Programs | Diverse range of adults responded to the intervention and was effective in lowering depression symptom severity scores | Outcome |
| 56 | Seangpraw et al. (2019) | Journal Article | Thailand | Evaluate the dietary behavior modification program Dietary Approaches to Stop Hypertension (DASH) with self-efficacy to reduce the risk of hypertension among the elderly as well as to motivate elderly people to have healthy dietary behaviors in order to prevent hypertension | Quasi-experimental (pre-post-test) | No justification for rural setting | Dietary education group session, training on improved healthy lifestyle (diet, physical activity) | Education and Training | Improved perceived severity, self-efficacy and preventive behaviors; no actual changes in anthropometic measurements, physical activity and smoking levels | Outcome |
| 57 | Shreffler-Grant et al. (2018) | Journal Article | United States | The purpose is to describe a feasibility study of a skill-building intervention to enhance health literacy about complementary and alternative (CAM) therapies among older rural adults and share lessons learned. | Quasi-experimental (Pre-post feasibility) | The independent nature of rural dwellers, scarcity of rural health care resources, prevalence of chronic health conditions among older rural adults, a general lack of knowledge about complementary and alternative medicines (CAM), and concern with the quality of available information about CAM make it critical that older rural consumers have sufficient health literacy about CAM. | An intervention to enhance complementary and alternative therapies health literacy; modules were presented face to face and by webinar | Education and Training | Intervention was implemented and evaluation and the team are exploring implementing it to other communities | Process |
| 58 | Smith et al. (2017) | Journal Article | United States | Work with local rural organizations to develop an evidence-based hatha yoga program intended to improve core strength and balance to reduce falls risk. | Quasi-experimental (Single-arm pilot study) | No justification for rural setting | Yoga (classes and at home) | Exercise and Physical Activity | Intervention was acceptable | Process |
| 59 | Sowle (2015)* | Dissertation/Thesis | United States | Evaluate the Living (well through) Intergenerational Fitness and Exercise (LIFE) Program in rural Iowa counties | Quasi-experimental | Older rural adults have higher rates of chronic disease, are further from health/fitness resources, are less active but also prefer programs that are free, accessible, fun and social | Physical, social, emotional, intellectual and vocational program | Health Promotion Programs | Increased physical activity, improved self-efficacy, well-received | Outcomes & process |
| 60 | Takada et al. (2018) | Journal Article | Japan | To determine the effect of an oral self-care program on oral, cognitive, and daily performance functions in rural community-dwelling older people with mild cognitive impairment. | Quasi-experimental | Dementia is concerning especially with there are limited social/economic resources so preventive interventions are needed | Oral self-care program | Health Promotion Programs | Programme may be an effective means to delay oral, physical, and cognitive decline in rural, community-dwelling older people with mild cognitive impairment | Outcome |
| 61 | Téllez-Rojo et al. (2013) | Meeting abstract | Mexico | Examine the nutritional impact of a non-contributory pension programme among older people in rural areas to improve the welfare of the elderly | Quasi-experimental (regression discontinuity) | Elderly people in Mexico experience poverty with limited access to social protection systems, making this population vulnerable, particularly for those who live in rural areas | Pension | Community Service (Pension) | Significant effect on protein, carbohydrate intake. No impact on BMI | Outcome |
| 62 | Theeke et al. (2015)* | Journal Article | United States | Present the initial feasibility and acceptability of LISTEN (Loneliness Intervention using Story Theory to Enhance Nursing-sensitive outcomes), a new intervention for loneliness | Randomized controlled trial | No justification for rural setting | Loneliness Intervention using Story Theory to Enhance Nursing-sensitive outcomes, a new intervention for loneliness | Health Promotion Programs | LISTEN was evaluated as feasible to deliver with no attrition and near perfect attendance; Participants ranked LISTEN as highly acceptable for diminishing loneliness with participants requesting a continuation of the program or development of additional sessions | Process |
| 63 | Volandes et al. (2011) | Journal Article | United States | Evaluate the end-of-life preferences of elderly patients in rural communities and whether preferences are associated with level of health literacy. | Randomized controlled trial | No prior studies on end-of-life decision-making have assessed the effects of health literacy in rural populations or the use of a video decision aid to better inform rural patients | Verbal description follows by video decision aid to assist education/decision making around advanced dementia care | Education and Training | People with higher health literacy referred comfort care, as did people who viewed the video decision aid | Outcome |
| 64 | Walters et al. (2017) | Journal Article | Netherlands | Determine the short- and medium-term effects of an intervention to support workers in providing preventive activities for older adults | Quasi-experimental | No justification for rural setting | Training program for preventive health problem-activities performed by home health care professionals | Education and Training | Did not significantly improve outcomes; however, there is potential that health promotion could be effective with enhanced delivery | Outcome |
| 65 | Wang et al. (2013) | Journal Article | Taiwan | To promote medication safety among rural elders with chronic illnesses | Randomized controlled trial | Challenge for rural health professionals to promote medication safety among older adults taking multiple medications | Volunteer coaching program | Education and Training | the volunteer coaching group improved their knowledge of medication safety; no change in attitude after the two-month study period; the group demonstrated three improved medication safety behaviors compared to the routine care group; volunteer coaching program and instructions with pictorial aids can provide a reference for community health professionals who wish to improve the medication safety of chronically ill elders | Outcome |
| 66 | West et al. (2010) | Journal Article | United States | Describe the use of telemedicine for setting goals for behavior change and examine the success in achieving these goals in rural underserved older adults with diabetes | Descriptive (based on another study which is an RCT) | Many rural adults with diabetes have limited access to diabetes educators; telemedicine is a feasible and acceptable approach to providing services. | Televisits with diabetes educator | Telehealth | Most behavioral goals were improved on or met | Outcome |
| 67 | Xu et al. (2020) | Journal Article | China | Investigate whether an integrated health care intervention improved health-related quality of life (HRQoL) and well-being among older patients with hypertension in rural China | Randomized controlled trial | Rural China's healthcare system is hospital-centered and fragmented, leading to poor continuity of care | integrated health care intervention | Health Promotion Programs | Decrease in reports of anxiety/depression; patients’ health awareness and knowledge improved; rate of compliance with a healthy lifestyle was low | Outcome |

**Literature 1B Summary Table**

|  | **Authors & Year** | **Type** | **Country** | **Methods** | **Concept** | **Context** | **Outcomes** | **Type of Evaluation** | **Typology of Intervention** |
| --- | --- | --- | --- | --- | --- | --- | --- | --- | --- |
| 68 | Agha et al. (2015) | Journal Article | Canada | Qualitative | Exercise programs designed to promote healthy ageing and reduce falls, delivered through DVD | Rural areas communities have shortage of health professionals, including physical therapists, the exercise program requires multiple visits by physical therapists; participants were asked about their experience of DVD-delivered Otago Exercise Program | Participants found the program useful, though it requires preparation and needs to be integrated in everyday activities | Process | Exercise and Physical Activity |
| 69 | Baker et al. (2017) | Journal Article | Australia | Qualitative (action research) | Improving confidence and independence in using Information and Communications Technology (ICT) (mobile devices and support), resulting in improved social engagement | Older adults are disadvantaged with the ICT use | Need more support and has to travel to main town for support, geographic signal reception, expensive and affordability of mobile data; no experience of ICT use; however they also gained confidence, independence and social engagement from ICT use. Participants were given mobile devices and support. | Outcomes & process | Telehealth |
| 70 | Barrera et al. (2017) | Journal Article | United States | Quantitative | Telephone delivered, modular, cognitive behavioral therapy (CBT) intervention for both late-life depression and anxiety for rual, homebound Veterans | No justification for rural setting | Showed improvement in depression and/or anxiety symptoms (note only descriptive statistics were used) | Outcomes & process | Telehealth |
| 71 | Batsis et al. (2020) | Journal Article | United States | Qualitative | Explores acceptability and value of telehealth-delivered intervention for obesity | Older adults with obesity living in rural areas are at high risk of decline | Acceptable and positive response from older people and clinicians | Process | Health Promotion Programs |
| 72 | Blocker (2019)* | Dissertation/Thesis | United States | Mixed-Methods | 10-week community-based education and exercise intervention program | Rural Americans (RA) have poorer vascular health and physical activity levels than their urban counterparts; dementia risk reduction among rural individuals requires a tailored approach | Healthy lifestyle outcomes significantly improved for education & exercise group; other outcomes were not significantly improved for the education & exercise group compared to control or education-only group | Outcomes | Exercise and Physical Activity |
| 73 | Boise et al. (2010) | Journal Article | United States | Mixed-Methods | Increasing screening and diagnosis of dementia | Improving dementia care is particularly challenging in rural areas where access to community resources, including medical specialists, are limited and primary care workloads are greater | Results included a substantial increase in screening for dementia, a modest increase in the proportion of patients who were diagnosed with dementia or mild cognitive impairment, and improved clinician confidence in diagnosing dementia | Outcomes | Health Promotion Programs |
| 74 | Conn et al. (2013) | Journal Article | Canada | Mixed-Methods | Staff from a community psychogeriatric outreach service facilitated referrals to a geriatric psychiatrist | Residents of remote and rural areas have reduced access to healthcare and often experience poorer health outcomes | Program was rated as being highly valued across all modalities of evaluation. Members of the referring team believe that access to a geriatric psychiatrist has broadened the team’s knowledge base, its use of assessment tools, and increased their ability to better construct their patients’ treatment plans; barrier is resistance of patient family members and some family doctors to telemedicine. | Process | Telehealth |
| 75 | Dal Bello-Haas et al. (2014) | Journal Article | Canada | Mixed-Methods | Feasibility of a telehealth-delivered exercise intervention for older people with dementia | Few high-quality exercise intervention trials exist and telehealth is being used to improve the delivery and availability of healthcare services for individuals living in rural areas, including exercise | Has the potential to facilitate improved healthcare services for individuals with dementia and their caregivers | Process | Exercise and Physical Activity |
| 76 | Dattalo et al. (2017) | Journal Article | United States | Mixed-Methods | Explores the organizational readiness and implementation strategies used for health promotion among older people | Rural elderly populations bear disproportion burdens of chronic disease, yet lack access to health care services compared to urban elderly populations | Effective resources are (1) External Partnerships, (2) Agency Leadership Commitment, (3) Ongoing Source of Workshop Leaders, (4) Health Promotion Coordination Tasks Assigned to Specific Staff, (5) Organizational Stability, and (6) Change Team Engagement | Process | Health Promotion Programs |
| 77 | Duggleby et al. (2020a) | Journal Article | Canada | Qualitative | Explores factors that facilitate sustainability of a rural community health intervention | Rural communities have additional challenges to implementation and sustainability of programs as there is shortage of health professionals and health related resources | Factors to facilitate sustainability included organizational context (inner context) and facilitation (facilitator and facilitation processes) | Process | Health Promotion Programs |
| 78 | Duggleby et al. (2020b) | Journal Article | Canada | Mixed-Methods | Program involving visiting older people every 3-4 weeks | Many older persons live in rural communities and experience poor health with little support when transitioning from chronic illness management to palliative care | Positive outcome and increase satisfaction | Outcomes & process | Health Promotion Programs |
| 79 | Elder et al. (2016)* | Journal Article | United States | Mixed-Methods | 10-week distance group exercise program | Limited access to health care services in rural areas; physical activity can be partially dependent on access to safe, low-cost, inviting environments; limited opportunities for indoor physical activity | Intervention found to be valuable by participants; significant improvement in physical function | Outcomes & process | Exercise and Physical Activity |
| 80 | Ford et al. (2019) | Journal Article | England | Mixed-Methods | Intervention involved providing money, a support manual and meetings to create local, innovative solutions to improve the booking system and transport | Rural practices as classified by the Health and Social Care Information Centre; Primary care access can be challenging for older, rural, socio-economically disadvantaged populations | The intervention was appropriate and acceptable | Process | Health Promotion Programs |
| 81 | Gould et al. (2016) | Journal Article | Canada | Mixed-Methods | Shuttle bus for a long-term facility used to provide transport and also provided an opportunity for individuals to participate in social engagement | Rural living presents a number of challenges for older adults, and one of the primary challenges is transportation. | The two most successful outcomes of the program were the provision of social interactions and opportunities for shopping. Although most individuals travelled on the shuttle bus alone, most participants engaged with others during the trips | Outcomes | Community Services (Transportation) |
| 82 | Kennedy et al. (2021) | Journal Article | United States | Qualitative | Framework for a multicomponent intervention delivered by staff for older people at risk of cognitive/functional decline due to impairments in cognition, mood or mobility | Rural communities need access to effective interventions that can prevent functional decline among a growing population of older adults | Targeting shared risk factors addressing social determinants of health through research support | Process | Health Promotion Programs |
| 83 | Keranović et al. (2013) | Journal Article | Croatia | Qualitative | Satisfaction and quality of a remote monitoring device for chronically ill patients | Rural resources are scarce | Patients discussed the feeling of security, possibility of quick intervention if needed, they could substantially save on transportation. | Process | Telehealth |
| 84 | McLoughlin et al. (2019) | Poster | Ireland | Quantitative | Rehabilitation program delivered in-home by multidisciplinary team | The program serves a predominantly rural catchment area where 14.5% of population were >65 years (compared to 11% in national average) | Intervention decreased hospital stay, facilitated independent living and identified needs (note that only descriptive statistics were reported) | Outcome | Health Promotion Programs |
| 85 | Meisner et al. (2019) | Journal Article | Canada | Qualitative | Explores the barriers and facilitators for a group leisure education program | Communities ranged between 1000-8000 citizens and were 90-110km from the nearest urban center; high proportion of older people in setting | Barriers included later life complexities (e.g., functional ability, responsibilities or willingness) affecting participation in programs, attitudes towards leisure activities, lack of confidence to try something new, embarrassed to impose on others, self-conscious in public and social settings, accessibility, receiving communication/information, cost; facilitators included meaningful experiences, attitudes around improving health (physical, mental, cognitive) | Process | Health Promotion Programs |
| 86 | Naik et al. (2012)* | Journal Article | United States | Mixed-Methods | Telehealth & diabetes (disease-specific) | Treatment options for diabetes and depression are rarely available for patients in rural settings | Participants responded positively to the collaborative goal setting and action-planning; clinically significant improvements observed to outcomes relating to diabetes and depression | Outcomes & process | Telehealth |
| 87 | Ohta et al. (2021)* | Journal Article | Japan | Mixed-Methods | Social cognitive theory (SCT)-based educational intervention | People in rural areas tend to have lower health literacy skills, social norms mean that people consider living without help virtue (stronger among older people) | Social cognitive theory-based educational interventions can positively impact self-efficacy in healthcare participation | Outcomes | Education and Training |
| 88 | O'Shaughnessy et al. (2011) | Journal Article | Ireland | Mixed-Methods | Rural transportation | Peripheral rural communities experience erosion of essential public services, leading to increase in mutual self-help initiatives (especially in agriculture, community development and financial savings). As services are more concentrated in larger centers of population, rural areas require car ownership to access these services. People who live rurally but do not have cars are cut off from this range of services. | Older people benefit from rural transportation system from social enterprise | Process | Community Services (Transportation) |
| 89 | Paddick et al. (2020) | Journal Article | Tanzania | Quantitative | Door dementia screening (using a mobile app) by rural primary health workers | Mobile health interventions form part of current WHO recommendations for non-communicable diseases in low-resource settings; in rural community, access to qualified psychiatrists are scarce | High (95%) consent to screening; app had high sensitivity but lacked specificity | Outcomes | Telehealth |
| 90 | Paschoa & Ashton (2016)* | Journal Article | United States | Mixed-Methods | Exercise programs focused on reducing sedentary behavior; improved physical function and participants were satisfied with the program | Physical inactivity is higher among rural populations, this may be attributed to: 1) natural social networks of older adults may not reinforce an active lifestyle; 2) older adults fear exercising outside due to unsafe neighbourhoods or natural environments; 3) lack of knowledge of the importance of exercising to improve quality of life, health status, daily functioning, health care costs and knowledge on how to exercise safely | Improved knowledge and physical function; participants were satisfied with the program | Outcomes & process | Exercise and Physical Activity |
| 91 | Pelcastre-Villafuerte et al. (2017) | Journal Article | Mexico | Qualitative | Development of a comprehensive healthcare model, interculturally appropriate, designed to meet the needs of Mexican Indigenous older adults | Half of Indigenous peoples do not have access to basic utilities; historical discrimination and marginalization due to ethnicity combined with limited access to public healthcare resources; only 12.9% of people who speak an Indigenous language have access to healthcare coverage by a subsidiary | Findings identified that lack of early diagnosis/treatment tend to contribute to chronic health problems, this population is not covered by public health/social insurance health problems, geography limits accessibility of services and continuity of care, ethnicity is an added barrier to preventing access to available health care services, study highlights need for healthcare systems to address intercultural and participatory needs | Outcomes & process | Health Promotion Programs |
| 92 | Pepin et al. (2014) | Journal Article | United States | Quantitative | A model community-based outreach program designed to overcome barriers to screening and case identification of vulnerable older adults in psychiatric distress | Transportation challenges, stigma, lack of available trained staff, cost are barriers to mental health services; rural areas in New Hampshire have disproportionately higher growth rates of older adults compared to urban areas | The program was able to screen for participants with depressive symptoms and alcohol abuse | Process | Health Promotion Programs |
| 93 | Pesut et al. (2017)* | Journal Article | Canada | Mixed-Methods | Palliative care support delivered at home | Few available services to support rural older people receiving palliative care, even though they require more transition compared to urban residents; rural communities also have the capacity to provide high quality care as there are both personal and professional relationships co-existing | Intervention is a promising intervention to meet the needs of population | Outcomes & process | Health Promotion Programs |
| 94 | Rahmawati et al. (2015) | Journal Article | Indonesia | Qualitative | Explores the role of community health workers to support people with hypertension | Management of hypertension in developing countries is poor; in Indonesia, it's the most common chronic disease | Results found that community health workers played a prominent role as gatekeepers in health care in managing hypertension particularly with facilitating checks, physical exercise, peer support, referrals | Process | Health Promotion Programs |
| 95 | Rebello et al. (2017) | Journal Article | United States | Quantitative | Telepharmacy education/support | 78% of veterans in Maine lived in rural areas, and 2.1% in highly rural areas | Effective in decreasing acute care utilization within 30 days after hospital discharge | Outcome | Telehealth |
| 96 | Scronce et al. (2021) | Journal Article | United States | Quantitative | Analysis of an ongoing community-based fall prevention program; improvements in physical function but not balance confidence | Challenges to implementation of falls-prevention programs in rural areas include paucity of healthcare resources, limited availability of instructors, space, funding, transportation, health care providers | Improvements in physical function but not balance confidence | Outcomes | Exercise and Physical Activity |
| 97 | Shreffler-Grant et al. (2020) | Journal Article | United States | Quantitative | Health literacy intervention on complementary alternative medicine | Older adults in rural areas are likely to have lower health literacy, which can impact on health decisions and overall health | Short term increase in CAM health literacy but decreased over the longer term | Outcome | Education and Training |
| 98 | Sorocco et al. (2013) | Journal Article | United States | Quantitative | Telehealth | No justification for rural setting | Improvements in strength, social functioning, decreased caregiver burden and increased compliance with treatment plan (no inferential statistics given) | Outcome | Telehealth |
| 99 | Sowle (2015)* | Dissertation/Thesis | United States | Mixed-Methods | Physical, social, emotional, intellectual and vocational program | Older rural adults have higher rates of chronic disease, are further from health/fitness resources, are less active but also prefer programs that are free, accessible, fun and social | Increased physical activity, improved self-efficacy, well-received | Outcomes & process | Exercise and Physical Activity |
| 100 | Staniuliene et al. (2016) | Journal Article | Lithuania | Quantitative | Analyses and proposes the design for development of domestic help services to enable older rural people and improve quality of life | Changing family structures has increased the demand for domestic social services particularly in rural areas, although there is a lack of these services in rural areas | The study identified that cooperation between government and non-government organizations are necessary, furthermore, implementing domestic help services would enable older rural people to have access to necessary services and improve their quality of life | Outcomes & process | Health Promotion Programs |
| 101 | Strand (2012) | Dissertation/Thesis | United States | Mixed-Methods | Exergaming and wellness program run by trainers | Older adults are physically inactive, and in rural areas; there is limited availability of physical activity programs | Increased physical activity and perceived health were the most reported perceived positive changes; significant increases in physical activity participation were maintained among participants who were physically inactive at baseline; Best-liked features were physical activity and socialization | Outcomes & process | Exercise and Physical Activity |
| 102 | Theeke et al. (2015)* | Journal Article | United States | Mixed-Methods | Loneliness Intervention using Story Theory to Enhance Nursing-sensitive outcomes, a new intervention for loneliness | No justification for rural setting | LISTEN was evaluated as feasible to deliver with no attrition and near perfect attendance; Participants ranked LISTEN as highly acceptable for diminishing loneliness with participants requesting a continuation of the program or development of additional sessions | Process | Health Promotion Programs |
| 103 | Tomioka et al. (2012) | Journal Article | United States (Hawaii) | Quantitative | Exercise classes | No justification for rural setting | High satisfaction with program, improvements in physical function | Outcome | Exercise and Physical Activity |
| 104 | Towne et al. (2015) | Journal Article | United States | Quantitative | Analysis of a chronic disease self-management program, a matter of balance/volunteer lay leader and enhance fitness program | Older adults in rural areas tend to be more vulnerable, characterized by limited resources, limited health care providers or higher poverty rates | Measures the extent to which the programs reached vulnerable older adults. It also examines characteristics of communities offering one of these programs relative to those simultaneously offering two or all three programs. | Process | Health Promotion Programs |
| 105 | Tsartsara (2015) | Conference abstract | Belgium | Qualitative (case study) | Development of a community-based integrated care management model of older people living in rural areas | Need for regional policy coordination to scale up ongoing innovative solutions adapted to the regional context to reduce budgetary costs for elderly care at both regional and national levels | The model was developed and implemented with a range of health care providers and services | Process | Health Promotion Programs |
| 106 | Ward et al. (2020) | Journal Article | Canada | Qualitative | Explores facilitators of physical activity maintenance at a community-based group exercise at a clinical wellness facility | In rural areas, community-specific and individual level barriers are amplified, need for increased attention to the subjective experiences of aging (especially for social participation) | Facilitators included social connections, individual contextual factors and healthy aging. | Process | Exercise and Physical Activity |
| 107 | Washburn et al. (2014) | Journal Article | United States | Quantitative | Strength training exercise classes initially led by volunteers but training participants to lead the group gradually | Compared to urban areas, rural older adults have limited access to exercise programs; older adults cite time commitments, travel time, geographic proximity and lack of social support as barriers to engaging in physical activity | Counties with lay leaders were 8.3 times more likely to have continuing groups compared to counties not using lay leaders | Outcome | Exercise and Physical Activity |
| 108 | Weinert et al. (2012) | Journal Article | United States | Quantitative | Evaluation of a personal health record (My Health Companion© (MHC©) developed to help individuals track, maintain, and appropriately communicate health information | In rural areas, accessing quality health care is impacted by scarcity of providers, limited dissemination and application of up-to-date health care information, long distances, and dangerous travel conditions | The MHC contributed to enhanced perceived self-efficacy, suggesting that it helped in health maintenance activities and was recommended to others | Outcome | Health Promotion Programs |
| 109 | Whitelaw et al. (2013) | Journal Article | United Kingdom | Qualitative | A sustainable social enterprise "Older People for Older People" where older people support services for other older people (e.g. transport, care hubs, cafes, radio station, outreach services ITC training, friendship support, history/cultural projects) | The project "Older People for Older People" (O4O) arose from the cost and difficulty of providing services to increasing number of older people in rural/remote communities; a range of case studies appeared to support these gaps | This report found that sustainability required a complex mix of supportive inputs balanced with entrepreneurship and that the "success" of social enterprises need to be assessed in multifaceted terms | Process | Community Services (Social) |
| 110 | Wilkinson et al. (2013) | Journal Article | Canada | Qualitative | Volunteer-facilitated expressive arts program designed to contribute to the wellbeing of socially isolated rural seniors | Social isolation experienced by older peoples | Although the program was beneficial there were certain issues such as a clash of values, expectations not realized, personal boundaries crossed, physical environment interfering with the creative process | Prcoess | Community Services (Social) |
| 111 | Zhenmian et al. (2016) | Journal Article | Japan | Mixed-Methods | Small agricultural business to improve social/economic participation through cultivating and selling agricultural produce | Rapid aging in rural and remote areas; economically, older people can play a role in generating high value economic, social and environmental outcomes on agricultural land | Findings suggests that older people involved in the business considered their health and economic situation to be improved through participation in vegetable cultivation and sales | Outcome & process | Community Services (Social) |

**Literature 2**

*Note: (Anonymous) has been indicated for authors in the Authors & Year column where the name of the authors of the literature was not published*

|  | **Authors & Year** | **Title** | **Source** | **Country** | **Description of Initiative** | **Typology of Initiative** |
| --- | --- | --- | --- | --- | --- | --- |
| 112 | Afayee (2017) | Integrating primary and behavioral healthcare for older adults in rural communities | Aging Today | United States | University of Pittsburgh Medical Center program | Health and Wellness |
| 113 | Anonymous (2010a) | Day 16 Election Promises | The Times - Transcript | Canada | Transportation Department's roadside brush-cutting budget by more than 40 per cent in the next four years to improve driver safety, especially in rural areas n create a bursary program to match | Transportation Services |
| 114 | Anonymous (2010b) | Transportation Initiative | PVA Publications | United States | Veterans Transportation Services (VTS) | Transportation Services |
| 115 | Anonymous (2010c) | On Lok Hosts Conference to Explore Feasibility of PACE for Rural Seniors: Policy Conference Brings Together Leaders in Eldercare To Advance Innovative Care Model in Rural California | US Newswire | United States | Program of All-inclusive Care for the Elderly (PACE) for Rural Seniors | Health and Wellness |
| 116 | Anonymous (2011a) | Media Release: The Royal Australian and NZ College of Psychiatrists | MediaNet Press Release Wire | Australia | Medical Specialist Outreach Assistance Program (MSOAP) have sought to improve access to specialist services in rural and remote areas | Health and Wellness |
| 117 | Anonymous (2011b) | Government of Canada Supports Seniors in Saskatchewan | Marketwire | Canada | Fraud Awareness for Seniors Toolkit and Abuse Prevention and Response Network project | Health and Wellness |
| 118 | Anonymous (2011c) | Transport service opens up a whole new world | The Sligo Champion | Ireland | West Sligo Rural Transport Initiative | Transportation Services |
| 119 | Anonymous (2011d) | The Kaiser Permanent Community Fund at Northwest Health Foundation Gives $3 Million in Grants | Health & Beauty Close - Up | United States | Local community gardens | Community Projects involving older people |
| 120 | Anonymous (2011e) | RCAM garden program to help fight hunger | Sun Journal | United States | Rural Community Action Ministry Gardening Program provides hunger prevention services to elderly | Basic Needs: Health and Wellness; Community Projects |
| 121 | Anonymous (2011f) | Around the County | The Herald | United States | The Transportation Assistance Program, also known as TAP, provides transportation for older adults and people with disabilities who live in Snohomish County's rural areas | Transportation Services |
| 122 | Anonymous (2011g) | Initiative Connects Underserved to Information Highway | Aging News Alert | United States | Mobile apps for Communities Challenge | Technology |
| 123 | Anonymous (2012) | BRIEF: State helps fund area development projects | McClatchy - Tribune Business News | United States | Access to Home program to make accessibility improvements to 24 homes of elderly or disabled residents | Housing Programs |
| 124 | Anonymous (2013a) | Rural Health; New Rural Health Findings Reported from D.C. LoGiudice and Co-Authors | Health & Medicine Week | Australia | Model of care providing services (including home services, meals, transport, respite, personal care and advocacy) | Health and Wellness |
| 125 | Anonymous (2013b) | Campbell River, Sayward receive Age-friendly BC grants | Campbell River Mirror | Canada | Program funding | Financial support |
| 126 | Anonymous (2013c) | Cash infusion a life-changer | Guelph Tribune | Canada | Adult day programs (healthcare organisations) | Health and Wellness |
| 127 | Anonymous (2013d) | Clinical Research; Reports Summarize Clinical Pharmacy Study Results from Ernst-Moritz-Arndt University | Health & Medicine Week | Germany | Model of care providing services AGnES-practice assistant, pharmacist, general practitioner (GP) and adherence supporting strategies (using a medication reminder chart, medication compliance aid) | Health and Wellness |
| 128 | Anonymous (2013e) | Diet and Nutrition; Investigators from University of Putra Malaysia Zero in on Diet and Nutrition | Health & Medicine Week | Malaysia | Nutrition education package | Education and Training; Health and Wellness |
| 129 | Anonymous (2013f) | Youth, elders groups formed for peace, development in Chitral | Asianet-Pakistan | Pakistan | Groups | Community Projects involving older people |
| 130 | Anonymous (2013g) | Case Study of Senior Cohousing Development in a Rural Community | Aging Today | United States | senior cohousing in a rural community | Housing Programs |
| 131 | Anonymous (2014a) | A cutting-edge local project aimed at installing video | The Northern Daily Leader | Australia | Installing video conferencing in homes of older people | Technology |
| 132 | Anonymous (2014b) | Hamilton council approves age-friendly policy | Flamborough Review | Canada | Policy | Age-friendly policy |
| 133 | Anonymous (2014c) | MCF doles out the dough to area charities | Bracebridge Examiner | Canada | A exercise for older female cancer patients | Health and Wellness |
| 134 | Anonymous (2014d) | Seniors pilot project battles isolation | Bracebridge Examiner | Canada | Seniors Community Adviser project, and strength volunteer system | Volunteering |
| 135 | Anonymous (2014e) | $2 million helps support seniors to stay at home longer | North Thompson Journal | Canada | Better at Home program; how we can all better support seniors where they live | Housing Programs |
| 136 | Anonymous (2014f) | Norfolk telemedicine trial proving successful | British Journal of Healthcare Computing | Canada | Use of a video equipment at their bedside | Technology |
| 137 | Anonymous (2014g) | Education providers of social services in rural areas of South Bohemia and Pilsen region (Vzdelavani poskytovatelu socialnich slu eb ve venkovskuch oblastech Jihoceskeho a Plzenskeho kraje) | MENA Report | Czech Republic | Workforce training | Health and Wellness |
| 138 | Anonymous (2014h) | New funding for communities to tackle loneliness | The Driffield Times | United Kingdom | Community Friendly Buildings, a new Rural Action Yorkshire (RAY) project, will inspire and facilitate village halls | Infastructure upgrades |
| 139 | Anonymous (2014i) | United States: USDA Seeks Applications for Grants to Increase Economic Opportunity and Improve the Quality of Life in Rural Areas | MENA Report | United States | Keeping Seniors Home Program; WMCAP is also working on a regional job creation plan | Housing Programs |
| 140 | Anonymous (2014j) | Franklin County seniors get help with housing, repairs | Sun Journal | United States | Rural Development's 504 Loan and Grant program will help seniors to update their homes to conserve energy; make home improvement | Housing Programs |
| 141 | Anonymous (2014k) | Reps. Kind, DelBene and Pingree Introduce RIDE Act to Ensure Low-Income Seniors Have Rides to Their Medical Visits | Federal Information & News Dispatch, LLC | United States | Rides to and from their doctor's appointments | Health and Wellness |
| 142 | Anonymous (2014l) | DelBene, Pingree and Kind's RIDE Act to Ensure Low-Income Seniors Have Rides to Their Medical Visits | Federal Information & News Dispatch, LLC | United States | Cost-effective medical transportation | Transportation Services |
| 143 | Anonymous (2015a) | Experts suggest measures - Drinking water for Thar | Asianet-Pakistan | Pakistan | Safe drinking water | Basic Needs: Health and Wellness |
| 144 | Anonymous (2015b) | Media Release: Victorian Taxi Association | MediaNet Press Release Wire | Australia | Multi-Purpose Taxi Program (MPTP) 600,000 taxi trips | Transportation Services |
| 145 | Anonymous (2015c) | Rehabilitation; New Rehabilitation Study Findings Have Been Reported by Researchers at Dalhousie University (Examining an occupational perspective in a rural Canadian age-friendly consultation process) | Health & Medicine Week | Canada | Consultation of Rural community | Health and Wellness |
| 146 | Anonymous (2015d) | Grants help communities take action against elder abuse | M2 Presswire | Canada | Community Action for Healthy Relationships Network | Community Projects involving older people |
| 147 | Anonymous (2015e) | Age-friendly plans | The News | Canada | Accessibility 2024 project to enhance the quality of life of local seniors, and are among 28 B.C. communities | Health and Wellness |
| 148 | Anonymous (2015f) | Rural bus service is vital for area | The Drogheda Independent | Ireland | Transport: Louth Public Participation Network, state agencies and other publicly funded bodies on a matter of vital importance to the rural community | Transportation Services |
| 149 | Anonymous (2015g) | Clinical Research; Research Conducted at College of Applied Science Has Provided New Information about Clinical Trials and Studies (Reablement in community-dwelling older adults: a randomised controlled trial) | Health & Medicine Week | Norway | Multi-component home based rehab | Health and Wellness |
| 150 | Anonymous (2015h) | Theatre group is 'opening minds to endless possibilities' | Coleraine Times | United Kingdom | Number of engaging activities | Community Projects involving older people |
| 151 | Anonymous (2015i) | Following His Urging, Brown Announces That VA Will Change Mileage Requirements for Veterans Seeking Medical Care | Federal Information & News Dispatch, LLC | United States | Non-Veterans Affairs health care facility | Health and Wellness |
| 152 | Anonymous (2016a) | Aust'n software to remotely diagnose eye conditions wins prestigious Google grant | Xinhua News Agency - CEIS | Australia | Vision at Home project | Health and Wellness |
| 153 | Anonymous (2016b) | Parish aims to share wisdom across the ages | West Carleton Review | Canada | The community of Carp to create opportunities for people of all generations to develop relationships | Community Projects involving older people |
| 154 | Anonymous (2016c) | Avondhu Blackwater Partnership 'Community Connect' service | The Corkman | Ireland | Community Connect service delivered by Avondhu Blackwater Partnership is a Community Service Programme (CSP) providing necessary services to older people who may feel lonely or isolated, especially those who live in rural and remote communities in North and East Cork and West Waterford | Health and Wellness |
| 155 | Anonymous (2016d) | TOKYO REPORT: Restaurants Racking Brains for Recruitment | Jiji Press English News Service | Japan | Work opportunities for older people in Restaurants | Paid Employment Programs |
| 156 | Anonymous (2016e) | Gloucester City Council [Edition 3] | The Gloucestershire Echo | United Kingdom | In Touch project, a project which enables older people to access health related and social activities in their communities. | Health and Wellness |
| 157 | Anonymous (2016f) | On Maine Tax Day, King Calls on IRS to Make It Easier for Elderly and Rural Mainers to File Taxes | Federal Information & News Dispatch, LLC | United States | Allows taxpayers to place orders for hard copy forms online | Technology for Community services |
| 158 | Anonymous (2017a) | Free travel pass has limited benefit, says study | Irish Times | Ireland | Transport | Transportation Services |
| 159 | Anonymous (2017b) | Snow removal projects help elderly | The Japan News | Japan | Help primarily elderly residents in rural areas remove snow on and around their houses | Basic Needs: Community Projects |
| 160 | Anonymous (2017c) | Senior Planet takes tech education to the stratosphere | Aging Today | United States | Fitness/wellness training program | Health and Wellness |
| 161 | Anonymous (2017d) | Learn to live better with extra help | Area News | United States | Information, equipment, in-home and community services, and support for carers. | Health and Wellness |
| 162 | Anonymous (2017e) | From affordable internet to video teller machines: ETA paper highlights outreach to the financially underserved | ATM Marketplace News Features | United States | Mobile banking services and updates | Technology for Community services |
| 163 | Anonymous (2018a) | Stay healthy, stay at home | Maitland Mercury | Australia | Wellness for Independence programs | Health and Wellness |
| 164 | Anonymous (2018b) | Lonely No More program to benefit socially-isolated seniors | Listowel Banner | Canada | Lonely No More plans to use teleconference calls to facilitate elder circles of peer support | Health and Wellness |
| 165 | Baker (2010) | Spending focus on front-line services: Council tax up less than Pounds 20 this year. Where does the money go? | Essex Chronicle | United Kingdom | Community learning | Education and Training; Community Projects |
| 166 | Bando et al. (2016) | The Effects of Non-Contributory Pensions on Material and Subjective Well Being | National Bureau of Economic Research, Inc, NBER Working Papers: 22995 | Peru | Pension 65 program | Financial support |
| 167 | Benson et al. (2017) | Rural older adults hit hard by opioid epidemic | Aging Today | United States | Training, Education, Age-Tailored Treatment | Education and Training; Health and Wellness |
| 168 | Bloom (2019) | The History and Growth of the Rural PACE Center: Support for rural aging in place: charting the success of rural PACE centers | Generations | United States | PACE Care Model to remain at home | Health and Wellness |
| 169 | By et al. (2015) | Graffiti comes of age as elderly Portuguese try urban art | Associated Press DBA Press Association | Portugal | Learning how to paint graffiti | Community Projects involving older people |
| 170 | Chen (2015) | Old-Age Pension and Intergenerational Living Arrangements | Federal Reserve Bank of St Louis | China | Pension program for rural residents | Financial support |
| 171 | Cholowsky (2016) | Council receives update from Viking/Beaver FCSS | The Weekly Review | Canada | Rural Elder Abuse Project, and Caregivers training | Health and Wellness |
| 172 | Coker (2015) | Mobile hearing center will fill gaps in rural West Alabama health care | The Tuscaloosa News | United States | Mobile hearing center | Health and Wellness |
| 173 | Coutre (2015) | Quick tips to make a home fall-proof | Star - News | United States | Falls Prevention Coalition in a recently announced a statewide initiative to help older people identify hazards | Health and Wellness |
| 112 | Crawford (2014) | Rural Fire Service helps reduce hazards at homes | South Coast Register | Australia | NSW Rural Fire Service's AIDER Program | Community Projects involving older people |
| 113 | Daily (2017) | Tech Savvy Elders Roadshow coming to Dubbo in March 2018 | Daily Liberal and Macquarie Advocate | Australia | Technical support | Technology for Community services |
| 114 | Dam et al. (2010) | Preventive palliation in the elderly - organizing health camps for the rural aged | Indian J Palliat Care | India | Providing palliative care to the rural-based elderly | Health and Wellness |
| 115 | De Mimos (2011) | Jewel Box | Rural Development Policy Projects Database | Portugal | Social Assistance through a community organisation/club | Community Projects to provide health and social services |
| 116 | Deery (2019) | Acorn SMART Tablet Pilot | Global Database of Age-friendly Practices | Ireland | Smart Device (tablet) to access health and community services | Technology for Health |
| 117 | Douglas (2011) | Campbell River to adapt to aging population | Campbell River Mirror | Canada | Infrastructure upgrades | Infrastructure upgrades |
| 118 | Doyle (2016) | New houses must be built for broadband | Irish Independent | Ireland | Service (internet/broadband) | Technology for Community services |
| 119 | Edwards (2010) | PACE Programs Find Success, Challenges in Rural Areas | Aging Today | United States | Serve seniors with chronic care needs in the community and keep them in their homes | Health and Wellness |
| 120 | El-Chantiry (2011) | Clean up the Capital in Constance Bay | West Carleton EMC | Canada | Snow Go Program, which assists seniors and persons with disabilities with their snow removal needs | Basic Needs: Community Projects |
| 121 | Elliott et al. (2015) | Developing a System Navigator Role in Primary Care Using a Co-Design Approach | International Journal of Integrated Care (IJIC) | Canada | System navigator can be developed and implemented in primary care | Health and Wellness |
| 122 | Faruk et al. (2017) | On green virtual clinics: A framework for extending health care services to rural communities in Sub-Saharan Africa | The Institute of Electrical and Electronics Engineers, Inc. (IEEE) | Africa | Virtual Clinics and integration of various health information systems such as Electronic/Mobile Health and Electronic Health Record systems | Technology for Health |
| 123 | Feingold (2018) | Proposal would prop up federal rental assistance program | New Hampshire Business Review | United States | Access to a federal rental assistance program | Housing Programs |
| 124 | Fennessy (2018) | Clare Cultural Companions – An Age & Opportunity Arts Initiative | Global Database of Age-friendly Practices | Ireland | Social activity focused on Arts | Community Projects to Connect and Socialise |
| 125 | Freitas (2012) | Remote Care Services in the Outermost Regions of Portugal | Rural Development Policy Projects Database | Portugal | ICT application to link users via an 'alarm button' | Technology for Health Services |
| 126 | Fuller (2011) | New class for Senior Leadership Program | Winston - Salem Journal | United States | Senior Leadership Enhancement Initiative; information on preventing fraud against the elderly, addressing workforce shortages, helping the aging stay in their homes, better serving the elderly in rural areas, and utilizing the elderly as volunteer resources | Community Projects involving older people |
| 127 | Gallagher (2014) | Drivers wanted in Harvey region | Daily Gleaner | United Kingdom | Driver program | Transportation Services |
| 128 | Gascon Herrero (2014) | Public transport, the key for tourism | Yearbook of the 'Gheorghe Zane' Institute of Economic Researches - JASSY / Anuarul Institutului de Cercetări Economice 'Gheorghe Zane' Iaşi | Romania | An INTERREG IV C project that study the situation of the transport and Best Practices in 12 mountain territories spread in 10 European countries taking in to account the environmental point of view of the practice | Transportation Services |
| 129 | Hast (2012) | Hello Päijät-Häme' - Friendly Call Service in Finland | Rural Development Policy Projects Database | Finland | Befriending Telephone services | Technology to Connect |
| 130 | Hogan (2012) | COMMUTERS and the elderly could soon be using the same bus as schoolchildren.: National | Irish Independent | Ireland | Rural Transport Bus | Technology for Community services |
| 131 | Israel et al. (2012) | The Value of Transportation for Improving the Quality of Life of the Rural Elderly | Federal Reserve Bank of St Louis | United States | Public transportation assessment | Transportation Services |
| 132 | Jaffe (2015) | Aging In Rural America | Health Affairs | United States | Programs to access to medical services, improve educational opportunities | Health and Wellness |
| 133 | Jesús-Azabal et al. (2021) | OPPNets and Rural Areas: An Opportunistic Solution for Remote Communications | Wireless Communications & Mobile Computing (Online) | Spain | Presence detection platform for elderly people who live alone and an analytic performance measurement system for livestock | Technology for Community services |
| 134 | Jones (2015) | The First Minister with his view from Cardiff: OPINION | Daily Post | United Kingdom | Improve access to care | Health and Wellness |
| 135 | Komar (2012) | Schwester Monika - Disabled Access Bus | Rural Development Policy Projects Database | Germany | Wheelchair-equipped van to allow transport | Transportation Services |
| 136 | Kröger (2014) | The Touring Stop | Rural Development Policy Projects Database | Finland | Community events such as workshops (basic skills), public lectures | Community Projects involving older people |
| 137 | La Poste (2021) | Trust your postman to look after your parents | La Poste | France | Postal Letter delivery to older parents | Community Projects to Connect |
| 138 | Lincs Bus (2021) | Rural transport services in the United Kingdom | Lincolnshire Country Council | United Kingdom | Transportation Services, Call Connect | Transportation Services |
| 139 | Magubane (2015) | New Hibiscus housing projects | The Mercury | South Africa | Housing renovation | Housing Programs |
| 140 | Marjoribanks (2018a) | Fighting to end loneliness: LINKAGE STIRLING Project aims to reduce isolation | Stirling Observer | United Kingdom | Pilot schemes and investment aimed at ensuring older people were not left isolated in their homes. | Health and Wellness |
| 141 | Marjoribanks (2018b) | Cash boost for the food train | Stirling Observer | United Kingdom | FoodTrain is a Scottish Charity | Basic Needs: Health and Wellness |
| 142 | Marnie Van (2020a) | New Life for Haiti welcomes 11 new trustees to its board of | Daily Herald | Haiti | Feeding programs | Basic Needs: Health and Wellness |
| 143 | Marnie Van (2020b) | New Life for Haiti hosts meal packing event | Daily Herald | Haiti | Feeding programs | Basic Needs: Health and Wellness |
| 144 | McGuire (2015) | Catering to diverse needs of adult learners: Aontas puts adults on an education path, whether for a career or the sake of learning | Irish Times | Ireland | Education programs for older people | Education and Training |
| 145 | Miller (2015) | Getting around when you no longer drive | Capital | United States | Door-to-door transportation services | Transportation Services |
| 146 | Murphy (2014) | 'To give and not receive' | TCA Regional News | United States | Partners in Ministry's Rural Outreach and Repair program can vary among paperwork, heavy lifting, and delivering wood to enable the elderly to heat those homes in a cold snap | Basic Needs: Health and Wellness; Community Projects |
| 147 | Okuyama et al. (2019) | Neighborhood environment and physical activity among rural Japanese older adults | European Journal of Public Health | Japan | Outdoor physical activity | Health and Wellness |
| 148 | Oliveira & Moreira (2013) | Improving and Extending a Mobile Home-Help Service (More and Better SMAD) | Rural Development Policy Projects Database | Portugal | Mobile Home-help service (home chores) | Technology for Home Services |
| 149 | P. R. Newswire (2016) | Easterseals to Help Communities Provide Accessible Transportation for Older Adults and People With Disabilities | Easterseals | United States | Accessible Transportation Community Initiative to increase options for independent mobility in several communities nationwide | Transportation Services |
| 150 | P. R. Newswire (2017) | American Diabetes Association® Receives Five-Year, CDC Cooperative Agreement to Establish a Multi-state Network of Sites to Address Prediabetes | American Diabetes Association | United States | The American Diabetes Association's multi-state network | Health and Wellness |
| 151 | Rapp et al. (2016) | The osteoporotic fracture prevention program in rural areas (OFRA): a protocol for a cluster-randomized health care fund driven intervention in a routine health care setting | BMC Musculoskeletal Disorders | Germany | The program consists of mobility and falls prevention classes, German Gymnastic Association | Health and Wellness |
| 152 | Roberts et al. (2016) | Growing the Dental Workforce to Serve Rural Communities: University of Washington's RIDE Program | Generations | United States | Health professional workforce (dental) | Health and Wellness |
| 153 | Rooney (2016) | Fossum attests to local care-home safety | 100 Mile House Free Press | Canada | Safety of residential care and volunteer work for advocates | Volunteering |
| 154 | Saskatchewan Seniors Mechanism (2021) | Saskatchewan Seniors Mechanism | Saskatchewan Seniors Mechanism | Canada | Programs for seniors which included fitness, gardening and social activities | Community Projects: Volunteering |
| 155 | Solorzano et al. (2018) | Home Tele-assistance System for Elderly or Disabled People in Rural Areas | The Institute of Electrical and Electronics Engineers, Inc. (IEEE) | Ecuador | Home Tele-assistance system, which can send personal information and geographical location through the network of elder or disabled people | Technology for Health |
| 156 | Taylor (2018) | Roadshow helps locals live full lives | Daily Liberal and Macquarie Advocate | Australia | Health and nursing services | Health and Wellness |
| 157 | The Associated Press (2014) | Okla. aging agency to award transportation grants | Associated Press DBA Press Association | United States | Public transportation services | Transportation Services |
| 158 | Thompson (2017) | The Senior Navigators Project helps maximize independence Project helps maximize independence | Chronicle - Herald | Canada | Senior navigators project (social isolation) | Health and Wellness |
| 159 | Thrower (2017) | BaptistCare boosts affordable housing stock | Goulburn Post | Australia | Affordable housing for older people | Housing Programs |
| 160 | Tsuji (2015) | Analysis of the long-run effect of e-Health intervention on chronic diseases: A DID-PSM approach | The Institute of Electrical and Electronics Engineers, Inc. (IEEE) | Japan | Effect of e-Health which transmits health data of elderly residents at home to the remote health center and monitors their health | Technology for Health |
| 161 | Ukkola (2012) | Dental Care Services Provided at Home | Rural Development Policy Projects Database | Finland | Mobile Dental care unit | Health and Wellness: Health Services |
| 162 | Wall (2014) | Partnership formed to bring dental hygiene to Seniors at Home | Arnprior EMC | Canada | Oral health | Health and Wellness |
| 163 | Woollhead & Mortensen (2012) | Village Meeting Point Idestrup | Rural Development Policy Projects Database | Denmark | Social Clubs (playing cards and meet-up) | Community Projects to Connect and Socialise |
| 164 | Zhang (2011) | Children, support in old age and social insurance in rural China | Federal Reserve Bank of St Louis | China | Rural Pension Program | Financial support |
| 165 | Zhou (2014) | Investigating Information Needs of Elderly People in Chinese Rural Community: Presenting an Information Service Framework | The Institute of Electrical and Electronics Engineers, Inc. (IEEE) | China | Information services - Public broadcasting services, extending the audiovisual collection, loaning audiovisuals, religious faith audiovisuals | Technology for Community services |
